# CPANNatNIC software for counter-propagation neural network to assist in read-across

**DOI:** 10.1186/s13321-017-0218-y

**Published:** 2017-05-22

**Authors:** Viktor Drgan, Špela Župerl, Marjan Vračko, Claudia Ileana Cappelli, Marjana Novič

**Affiliations:** 1 Department of Cheminformatics, National Institute of Chemistry, Hajdrihova 19, 1001 Ljubljana, Slovenia; 2 Laboratory of Environmental Chemistry and Toxicology, IRCCS-Istituto di Ricerche Farmacologiche Mario Negri, Via La Masa 19, Milan, Italy

**Keywords:** Counter-propagation neural network, Read-across, Software

## Abstract

**Background:**

CPANNatNIC is software for development of counter-propagation artificial neural network models. Besides the interface for training of a new neural network it also provides an interface for visualisation of the results which was developed to aid in interpretation of the results and to use the program as a tool for read-across.

**Results:**

The work presents the details of the program’s interface. Parts of the interface are presented and how they can be used. The examples provided show how the user can build a new model and view the results of predictions using the interface. Examples are given to show how the software may be used in read-across.

**Conclusions:**

CPANNatNIC provides a simple user interface for model development and visualisation. The interface implements options which may simplify read-across procedure. Statistical results show better prediction accuracy of read-across predictions than model predictions where similar compounds could be identified, which indicates the importance of using read-across and usefulness of the program.

**Electronic supplementary material:**

The online version of this article (doi:10.1186/s13321-017-0218-y) contains supplementary material, which is available to authorized users.

## Background

In the past several years, there is an increasing interest in using in silico tools for risk assessment of chemicals. The reasons for higher interest can be found in Registration, Evaluation, Authorisation and Restriction of Chemicals (REACH) legislation in European Union which requires registration of a large number of chemicals in use. The legislation allows using read-across for toxicity assessment under certain conditions written in the regulation. Definition of read-across and its correct use are still rather unclear. Patlewicz et al. [1] gathered several definitions of read-across from different sources [e.g. United States Environmental Protection Agency (US EPA), European Chemical Agency (ECHA), The Organisation for Economic Co-operation and Development (OECD)]. Concisely, we may understand the definitions of read-across as an approach to predict a property of a chemical based on the same property of one or more similar chemicals. Different tools already exist which can be used for read-across, for example OECD QSAR Toolbox [2], ToxRead [3], TEST [4] and VEGA [5].

In this paper we present a new tool which can be used for development of counter-propagation artificial neural network (CPANN) models. The models can be later used either for direct prediction of the endpoint under consideration for new, i.e. untested compounds, or for read-across approach. The software provides a graphical user interface which was designed to facilitate read-across based on analogue or category approach using CPANN models. CPANNs are particularly suitable for these approaches because of their ability to group compounds according to their structural similarity. Although the software was initially built to facilitate read-across for toxicity assessment of substances, its usage is not limited to toxicity-related endpoints since the user describes compounds in the input data file(s) which may include numerical values of any property.

### Basis for read-across

As mentioned above, the software uses CPANN models. The results of the predictions can be viewed in a simple graphical user interface with compounds placed on the map, called a “top-map”, according to their similarity which can be used as the basis for read-across predictions. The learning principles of Kohonen and CPANNs are well established and can be found in detail elsewhere [6–8]. Some definitions are given below so that the user can better understand the results produced by the software.

**Fig. 1:**
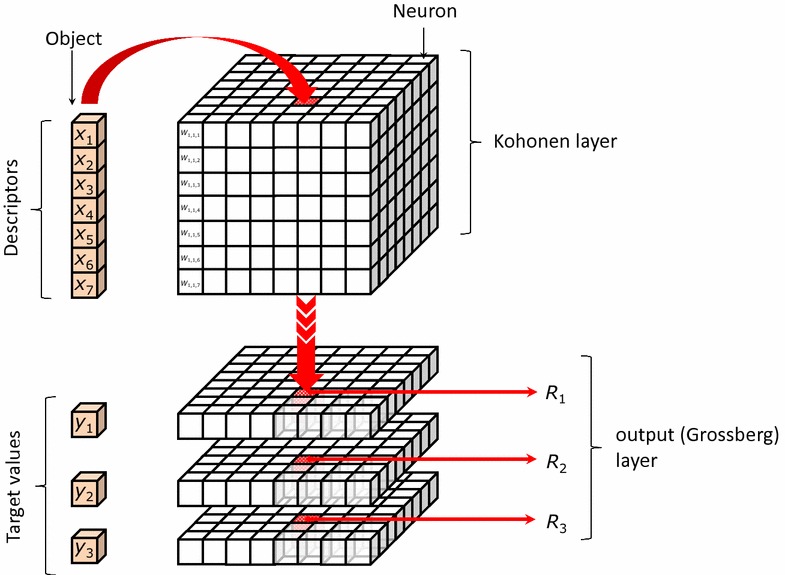
A scheme of counter-propagation neural network. An input object (vector of *X* values—descriptors) with descriptors (*top-left*) and endpoint values (*bottom-left*). CPANN scheme is shown on the *right*. Kohonen layer is on the *top* and the output layer with three response levels used to draw response surfaces is below the Kohonen layer

Schematic representation of a CPANN is shown in Fig. 1. It is composed of Kohonen layer and output (Grossberg) layer. It can be visualized as a 3D matrix of values called weights (*W*). One column (vector) of weights is called neuron. The figure schematically shows how the results of predictions (*R*_1_–*R*_3_) are obtained. First, the Euclidean distance between each neuron and the object is calculated using descriptor values and weights in Kohonen layer. Then the most similar neuron to the objects is identified as the neuron with the shortest Euclidean distance to the object, which is indicated on Fig. 1 with red colour. This neuron, excited by the object, is called “central neuron”. To get the predictions from the output layer, the position of the central neuron is projected onto the output layer and the results are read from the corresponding position for each target (property/endpoint). For each descriptor and target, a 2D surface plot can be obtained from the weights which is called “level plot”.

When all the training set and external set objects are tested one can obtain a top-map showing how the objects excited the neuron. The objects which are more close to each other are more structurally similar, and vice versa. This offers us a method which can be used for read-across; first similar compounds to our object are found and then experimental value of similar compounds can be used to predict property value of the selected compound.

The neurons shown in Fig. 1 will be represented in the graphical user interface of the software as squares containing the compounds which excited the neurons (i.e. a “top-map” will be shown). When “level plots” will be shown, each square will correspond to one weight in the selected level which corresponds to a descriptor or target (2D surface plot). The compounds can be represented by identification number, class label or as a 2D structure of the compound. The Euclidean distances which will be reported together with other information related with the predictions for objects are those Euclidean distances calculated between the object and the neuron.

## Implementation

The counter-propagation artificial neural network learning method presented in the article by Zupan et al. [8] was used for implementation. CPANNatNIC is entirely written in Java programming language. The program uses The Chemistry Development Kit (CDK) library (version 1.5.4) [9] for displaying 2D structures of compounds from SMILES strings. The program was written using NetBeans IDE 8.1 and Java JDK version 1.8 (64-bit).

### Installation

Java version 8 is needed to run CPANNatNIC software. The software can be freely downloaded from http://www.ki.si/fileadmin/user_upload/datoteke-L03/SOM_ver/v1_01/. The software is also available in Additional file 1 and its source files in Additional file 2. To install CPANNatNIC, unzip the downloaded file to a new folder. The folder will now contain two files. The file “CPANNatNIC.zip” contains all necessary files to run CPANNatNIC application and the file “example_input_data.zip” contains example input files. Unzip CPANNatNIC.zip file. The application “CPANNatNIC.jar” will be located in CPANNatNIC folder. To run the application, use command prompt and change current directory to the directory with the application and type *java*-*jar “CPANNatNIC.jar”*. Alternatively, you can double click “CPANNatNIC.jar” in case your operating system can execute “jar” files in this way.

### Limitations

The program was tested using Windows 7, 64-bit. Java 1.8 should be installed prior using the program. Successful execution of CPANNatNIC software is dependent on available Java heap memory. It is recommended that you have at least 8 GB of RAM installed on your computer. For example, you can allocate Java heap memory by executing command *java*-*Xmx4096m*-*jar “CPANNatNIC.jar”* to allocate 4 GB of Java heap memory for the application. There may be high memory requirements when saving large “top-maps” to PNG files, thus using smaller neuron sizes is preferred. Higher number of available processor cores may decrease the time needed to display 2D structures of compounds on the “top-map”. The recommended screen resolution is at least 1280 × 1024 pixels. The description given within this text presumes that the user uses standard “right-handed mouse” where left mouse button is used for primary click (a “click”) and the right mouse button is used for secondary click.

CPANN models are stored in text files where each column corresponds to a specific variable. When the user is using an existing model he/she should prepare an input file where the variables are stored in the same column order to obtain correct results. The software will produce warnings when the variable names in the input file are not the same as in the model file but will not stop the calculation.

### The program structure

**Fig. 2:**
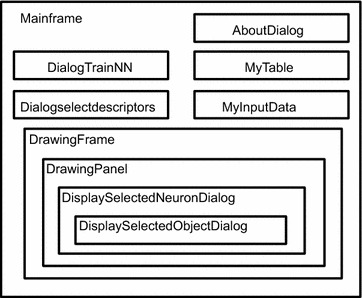
The main classes of the program and their hierarchy. Mainframe corresponds to the main window of CPANNatNIC and DrawingFrame to the user interface for graphical representation of the results

The main parts of the program were individually developed as Java classes. Figure 2 schematically shows hierarchy of these classes. The classes shown in Fig. 2 represent visible objects, such as frames, dialogs or panels. An exception is “MyInputData” class which is used for storing different variables used during program execution (e.g.: descriptor values, weights of CPANN model, variable names, predicted values for objects, position of excited neurons).

As shown in Fig. 2, the main class used is “Mainframe” which represents the main window of the application and is used mainly for model development. “AboutDialog” is used to show basic information about the program. “DialogTrainNN” is used for training of CPANN, “Dialogselectdescriptors” is used to select descriptors when performing predictions, and “MyTable” is used to show descriptor values or CPANN weights.

The main window of the interface which is used for displaying of the results represents “DrawingFrame” class. The “DrawingFrame” class uses “DrawingPanel” for displaying neurons of the top-map. “DisplaySelectedNeuronDialog” is used within “DrawingPanel” for displaying individual neurons and “DisplaySelectedObjectDialog” is used when displaying an individual compound.

### Graphical user interface

Before using the software, the data should be prepared in an appropriate format. The data which are required for each object are the values for independent variables (descriptors), dependent variables (targets), class and object identification number (object’s ID). A detailed description of input dataset files is given in the user guide provided with the application so that the user can manually prepare input files in the required format. An example of Excel file which can be exported to tab-delimited text file (Additional file 3) used as a data input file is included within the article as Additional file 4.

Graphical user interface consists of the main window which opens when the application starts and a window which is used for graphical representation of the results and becomes available when the results of predictions are available from the main window. The main window provides functionality of the software which can be used for the development of new CPANN models and provides access to the interface for graphical representation of results. The options available in both windows are described in the following sections.

**Fig. 3:**
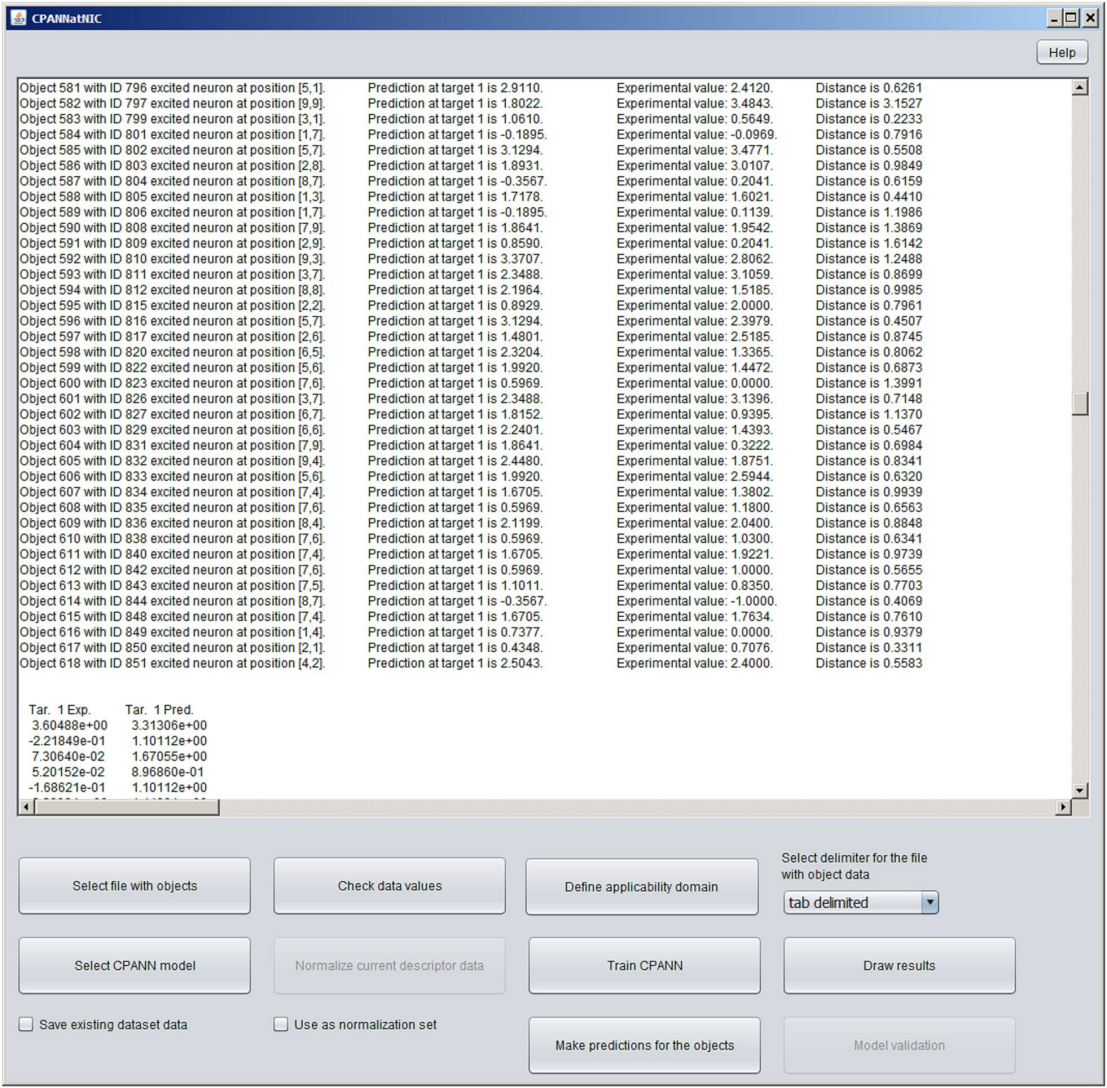
The main window of CPANNatNIC

The main window is shown in Fig. 3. The central area of the main window is a text area window which is used for displaying relevant information generated during program execution. The data which are displayed in the text area are related to the datasets and models read by the program, the results of the predictions made by the program and information regarding certain errors which may occur during program execution. When the program is started from command prompt, some additional information may be displayed in the command prompt or in a file in case the output is redirected to a file which can be then used as a log file (for example by using command *java*–*jar “CPANNatNIC”* > *logfile.txt*).

Below the text area, there are several options which become accessible when there are certain conditions fulfilled during program execution. For example, “Train CPANN” button will not be available until appropriate data are read from a dataset file. Importing data from an input file should be the first step after an appropriate delimiter, used in the file, is selected from drop-down menu.

**Fig. 4:**
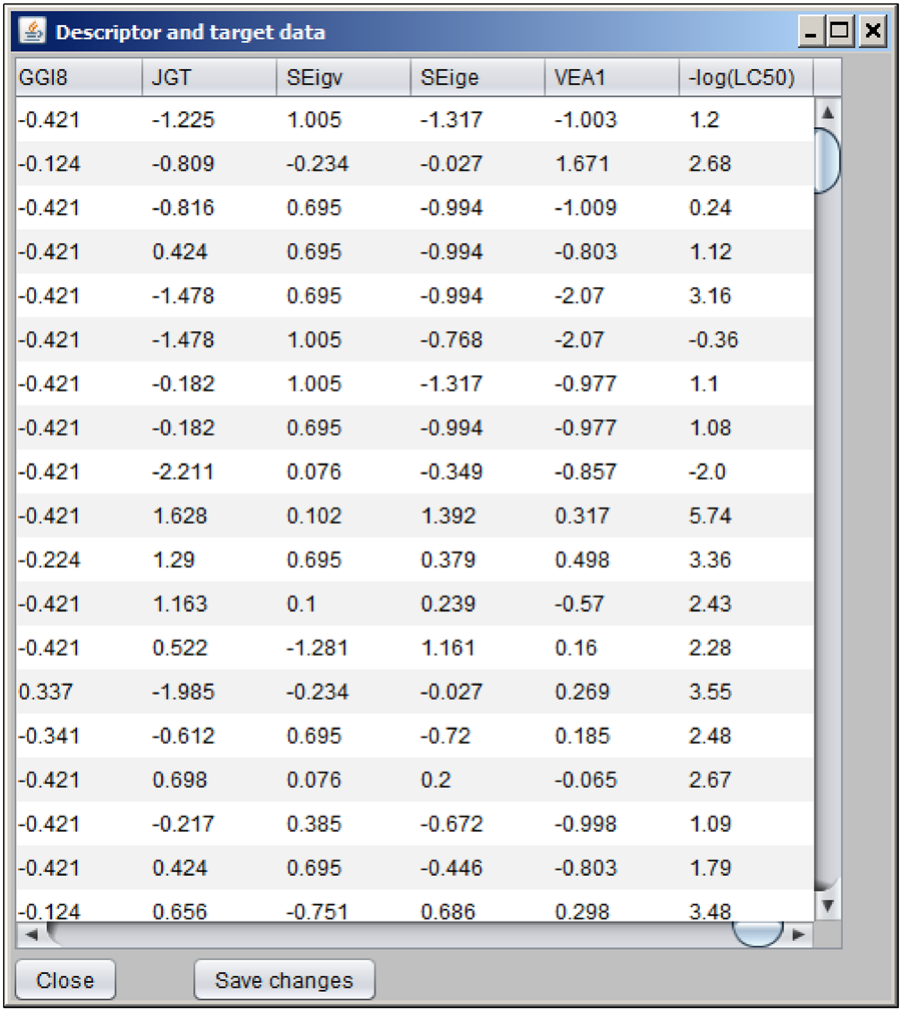
Table showing descriptor and target values

When the data are available in the program they can be viewed by clicking button “Check data values”. This will show a table similar to the one shown in Fig. 4. When a CPANN model is available, a similar table will appear also for the model that will display values of CPANN weights. Each line in the table represents an object in the same order as it appears in the input file and each column represents one variable (the names of the variables are written as column labels). If the dataset is training set, it can now be used to build a new model. If the dataset has not been normalized, the program can be used to normalize descriptor data. This is convenient if we have several datasets and they should all be normalized using the same normalization factors. When a new model is generated using training set data which were normalized using the software, the normalization factors are automatically saved into the model file and can be later used for normalization of new datasets. The normalization is done only for independent variables (descriptors) using Eq. (1).

1 $$ X_{normalized} = (X_{i} - X_{average} )/s $$

In Eq. (1), *X*_*normalized*_ represents normalized value of *X*_*i*_ which is the descriptor *X* of object *i*. *X*_*average*_ represents an average of all descriptor *X* values in the dataset used for training CPANN, and *s* is standard deviation of these values.

**Fig. 5:**
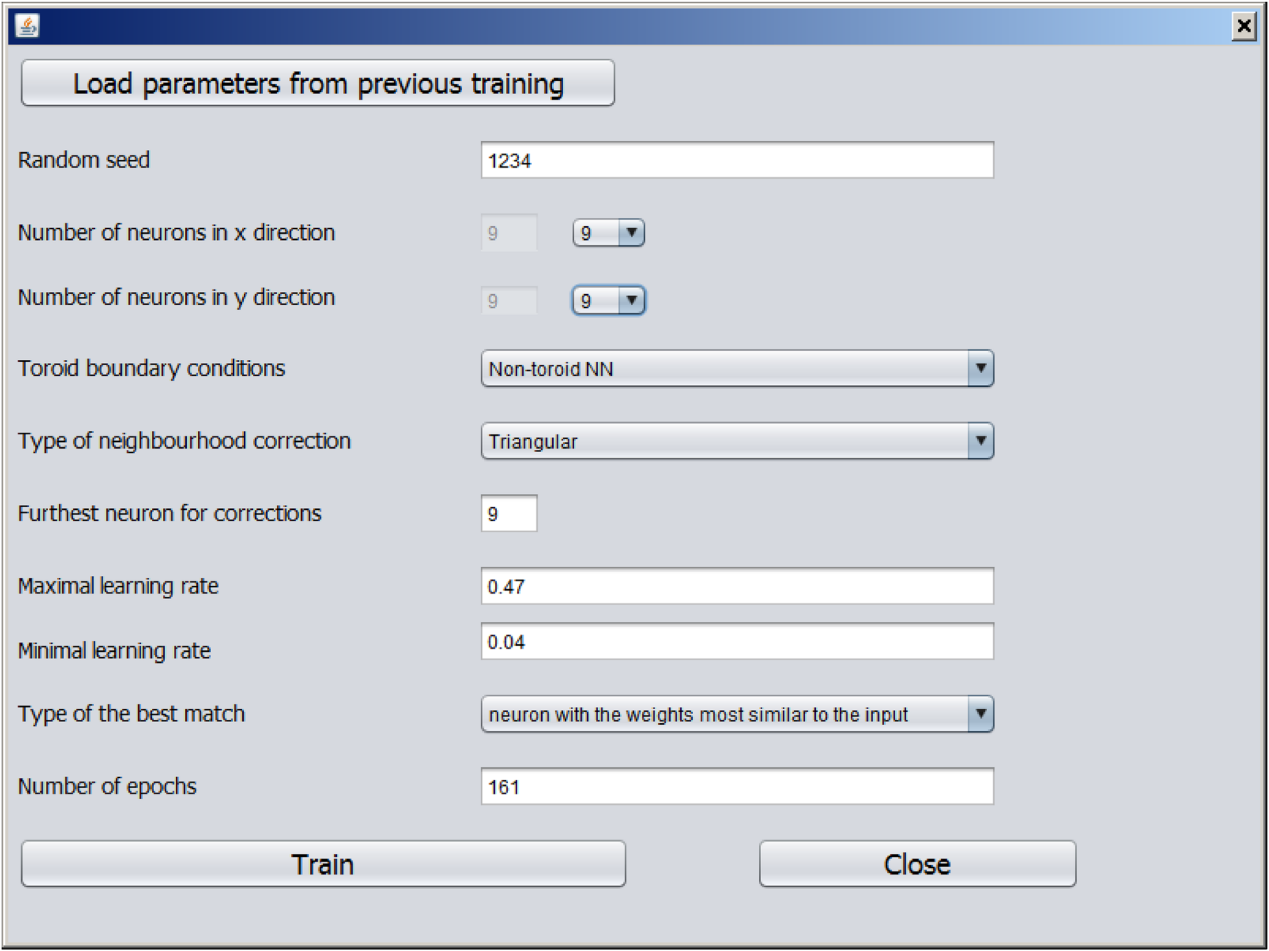
The dialog box used to define parameters needed for training of CPANN

**Fig. 6:**
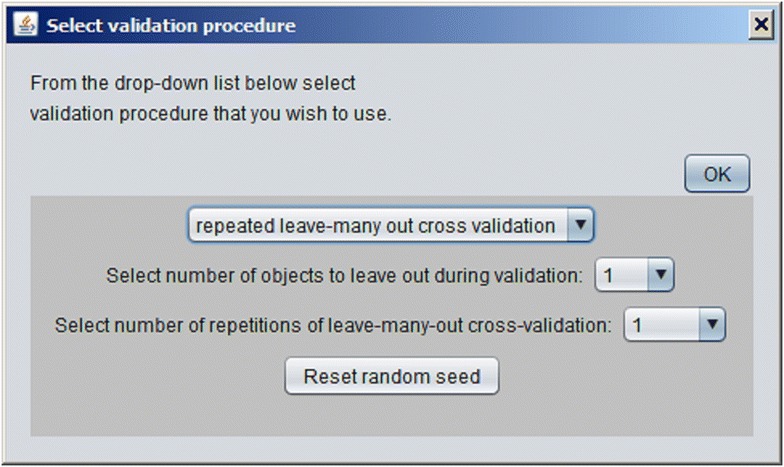
The dialog box for selection of a validation method

**Fig. 7:**
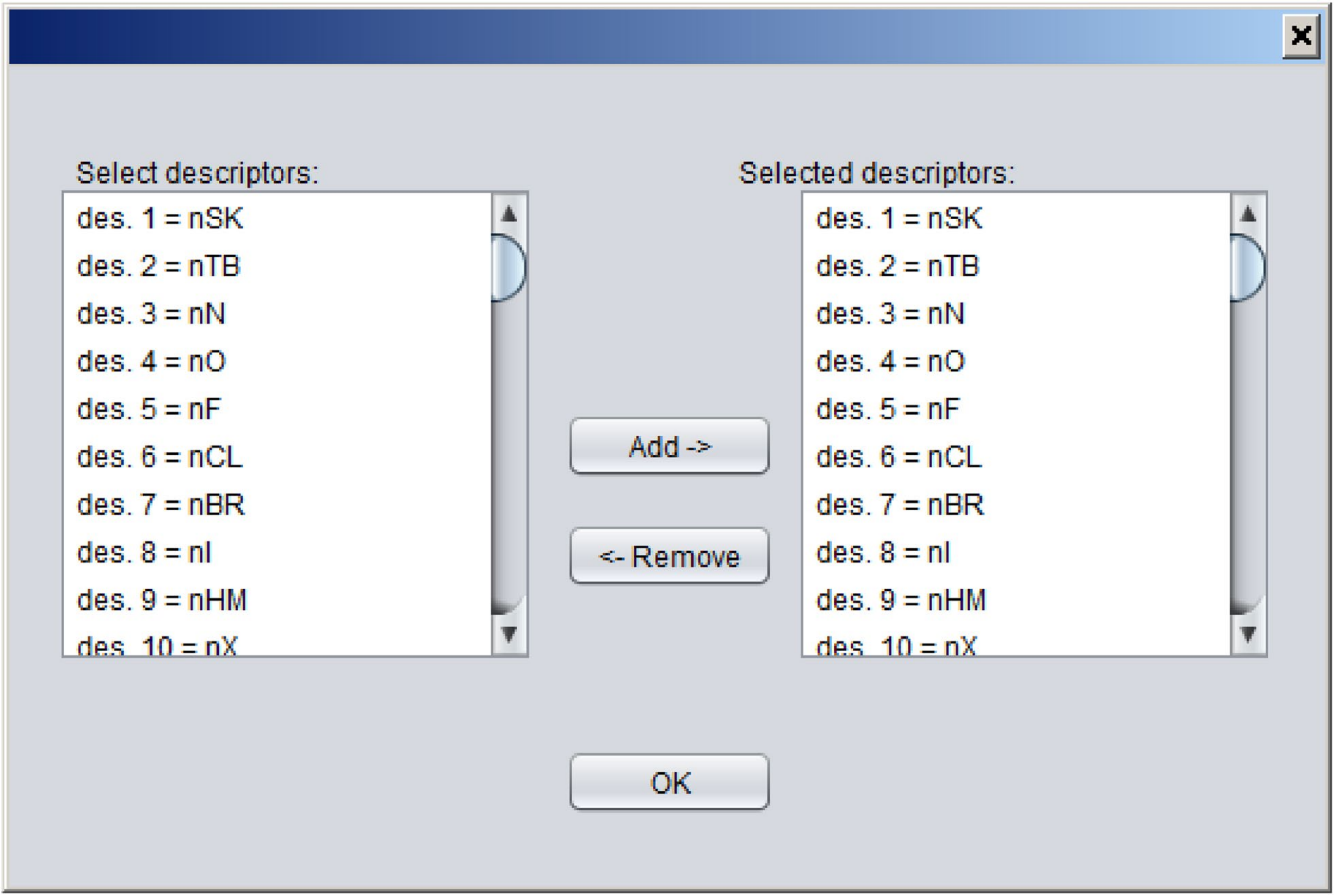
The dialog box for selecting descriptors before making predictions

**Fig. 8:**
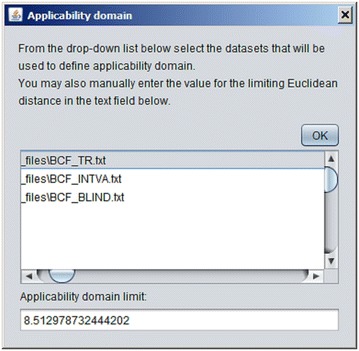
The dialog used to define applicability domain

A new model can be developed using imported dataset by pressing the button “Train CPANN”. A window, such as shown in Fig. 5, will appear with default values of the required parameters shown in the window. After the “Train” button is pressed CPANN training will start. When a model has been successfully generated model validation can be performed using button “Model validation” or predictions can be made for currently imported data using button “Make predictions for the objects”. In both cases, the results will be displayed in the text area of the main window. The results of the predictions will show for each object its identification number (ID), the neuron excited by the object, experimental value (the value written in the dataset file) and Euclidean distance of the object to the neuron. Additionally, information regarding root-mean-square error (*RMSE*) and correlation coefficient between experimental and predicted values will be given. Also, a textual representation of the top-map that is showing IDs or classes of the objects will be written. In the case of model validation, experimental and predicted property values, root-mean-square error of cross-validation (*RMSEcv*) and correlation coefficient of cross-validation (*Rcv*) will be reported. When the button “Model validation” is pressed a dialog, shown in Fig. 6, will open and the user may select between different options for model validation, such as: leave-one-out cross-validation, leave-many-out cross-validation, Y-scrambling, and repeated leave-many-out cross-validation. The procedures implemented for leave-one-out cross-validation and leave-many-out cross-validation keep the initial order of the training set object while the procedure for repeated leave-many-out cross-validation first shuffles the objects before each repetition and then performs leave-many-out cross-validation. When “Make predictions for the objects” button is clicked, a dialog box, such as the one in Fig. 7, will appear where the user may select the descriptors which are used to determine the position of the central neuron for all objects when making predictions. Usually, all the descriptors used during the training are selected. The user may change the selection to observe how different selection affects the grouping of objects. The button “Define applicability domain” becomes available after the predictions are made. When the user presses the button a dialog shown in Fig. 8 will appear where the user can select one or more datasets which can be used to define applicability domain. The applicability domain is defined according to the method proposed by Minovski et al. [10]. The objects with the Euclidean distance to the central neuron which is smaller or equal to the limiting Euclidean distance are within the applicability domain. The user may also manually enter the value which he/she considers as appropriate for the limiting Euclidean distance. When new predictions are made after the applicability domain is defined, then in the prediction results in the text area of the main window it will be also written whether the object is in applicability domain or not.

**Fig. 9:**
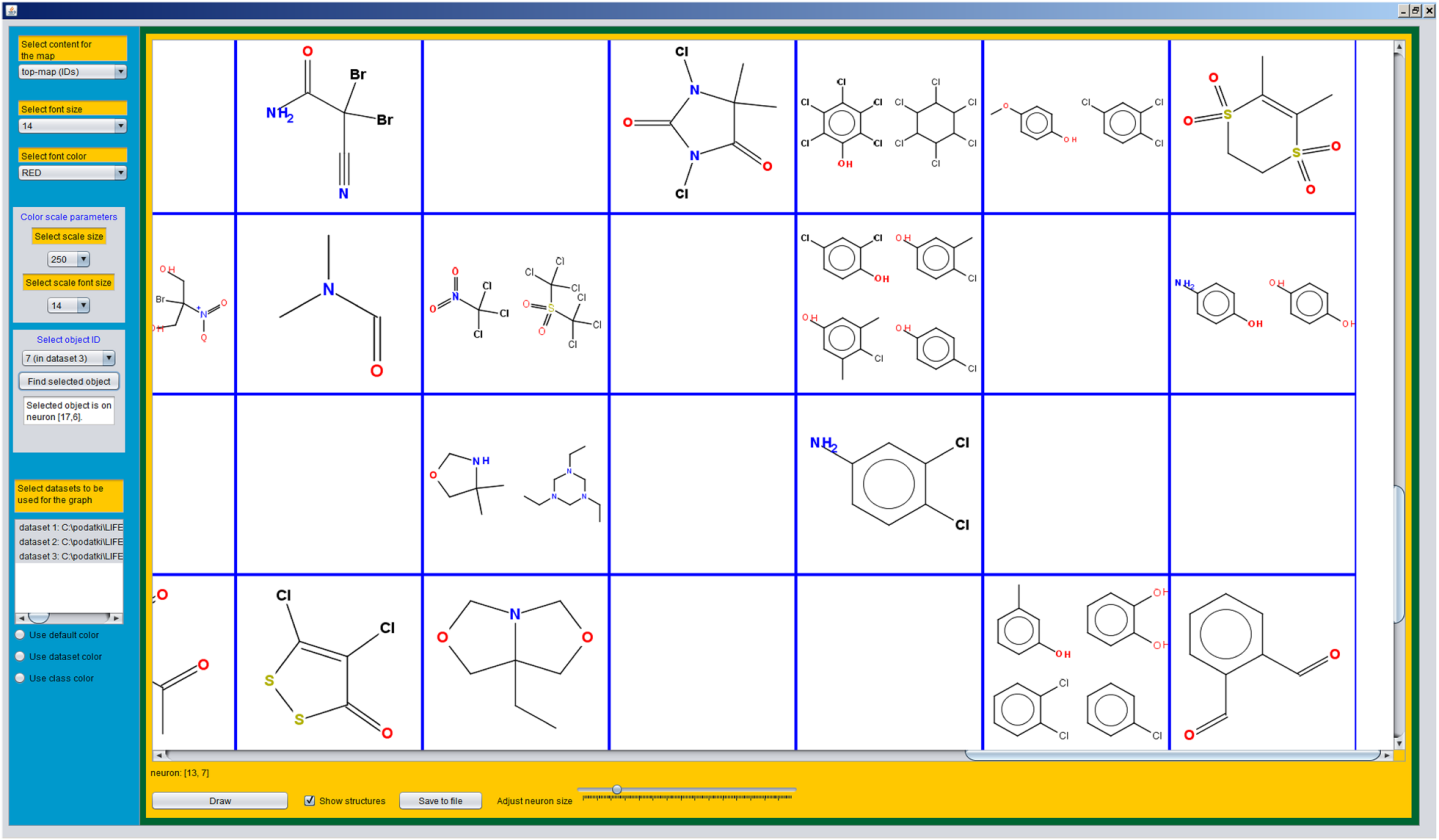
The interface for graphical representation of results showing a top-map

Some results of the predictions made using CPANN model can be viewed in a graphical user interface which is shown in Fig. 9 and can be accessed using button “Draw results” from the main window shown in Fig. 3.

A top-map will be graphically displayed when the button “Draw” is pressed. The options that affect the appearance of the results and the content shown are accessible from the blue panel in Fig. 9. Some functions are available using left and right mouse clicks on the neurons shown on the map.

The top-map will initially show ID numbers of the objects (compounds) that excited the neurons on the map. The datasets which are used to build the map can be selected from the list of datasets labelled as “Select datasets to be used for the graph”. Different colours can be defined for objects from different datasets or for objects belonging to different classes. This can be done using appropriate selection at the bottom of the blue panel which will open a new window where the user can define colours for datasets or classes. This may help to visually assess distribution of objects belonging to different classes or datasets.

Besides the presentation of ID numbers or classes the interface also supports displaying 2D structures of compounds on the map. To display 2D structures of compounds, “Show structures” check-box should be checked and a file containing a list of compounds’ ID numbers and corresponding smiles should be opened. The content shown on the map can be changed using drop-down menu labelled as “Select content for the map”. From the drop-down menu each descriptor and target level can be shown on a map as 2D surface which is coloured according to the weight values corresponding to the selected variable of the CPANN model. Classes or IDs of the compounds can be seen when “Show structures” check-box is not selected and the item “top-map (classes)” or “top-map (IDs)” is selected, respectively.

When there are many objects shown on the map, finding one particular object can be a tedious task. Thus, an option for locating an object on the map has been added. An object can be located by selecting the object’s ID from the drop-down menu labelled as “Select object ID” and then pressing the button “Find selected object”. The position of the neuron with the object will be shown in the text area below the button. A new window will appear that is showing the neuron which was excited by the object. The selected object shown on the neuron will be marked by a red rectangle. Any other neuron can also be shown in a new window by “double-clicking” on the desired neuron. Right-hand mouse button click on the object can be used to view any object shown on the neuron.

As mentioned before, CPANN training produces models which group similar objects close together on the top-map. This can be useful for the assessment of reliability of the prediction made for an object and also makes it possible to use the objects from neighbouring neurons for read-across. The interface gives the possibility to visually identify similar neurons using Euclidean distance or Tanimoto coefficient. Tanimoto coefficient is calculated using formula for continuous variables as reported in the literature [11, 12]. To visualize Euclidean distances or Tanimoto coefficient between neurons, the user should right-click on the neuron which should be compared to other neurons. A menu will appear with a few options on the list. The user can select “Show map of Euclidean distances to the selected neuron” or “Show map of Tanimoto similarity coefficients to the selected neuron” which will show a map of Euclidean distances or Tanimoto similarity coefficients between the selected neuron and the other neurons.

The map showing the Euclidean distances or Tanimoto coefficient can be saved by selecting “Save map of distances/similarities between neurons” from the menu, while the map showing the content selected from the dialog box can be saved using “Save to file” button below the map. When “Save to file” button is used, the program will also generate images of neurons in folder “resultingimages” representing neurons and “graphview.html” file for viewing the map in a web-browser. The files will be saved in the folder where the last input file was selected.

## Results and discussion

The functionality of the program described in the previous section can assist in read-across process. Two datasets will be used below to show how the program may be used for read-across. Here, it should be stressed that the models used for read-across are the same as the ones used to obtain model predictions. The examples will be shown using one pre-built model for prediction of acute toxicity towards rainbow trout (*Oncorhynchus mykiss*) and one example will show how a model can be built using bio-concentration factor. The models and datasets supporting the conclusions of this article are included within the article as additional files.

**Fig. 10:**
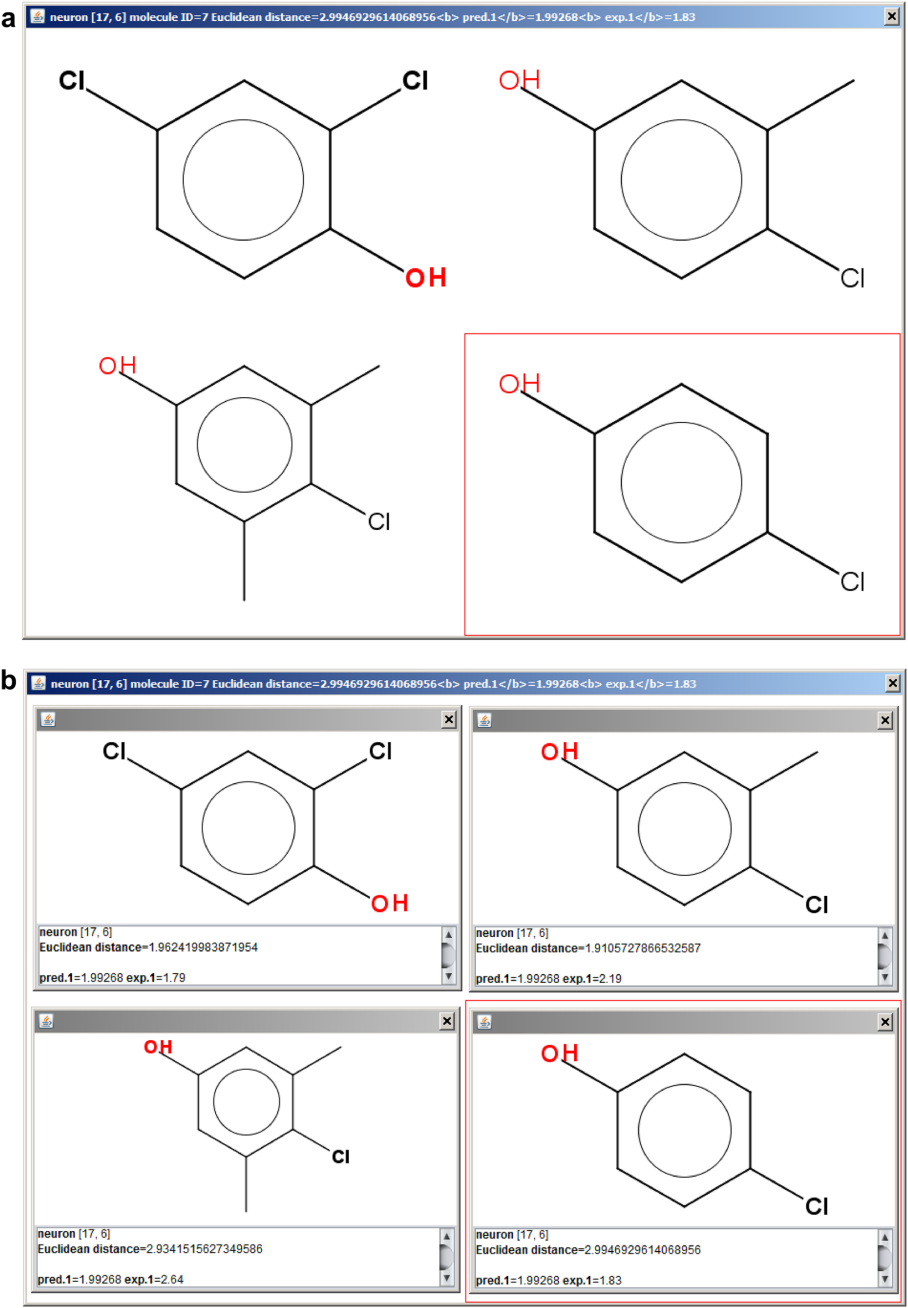
A neuron excited by different objects. The external set object with ID = 7 on **a** is surrounded by a *red rectangle*. The objects from training set and internal test set can be used for read-across. **b** The same objects in separate windows where information about the objects and model predictions can be found. Each of the windows can be opened by right-hand mouse button click (when using “right-handed mouse”) on the corresponding structures shown in **a**

As the first example, we show an example which requires smallest number of steps to obtain CPANN top-map that can be used for read-across assessment. In this example, we will use an existing model for acute toxicity which is available in the Additional file 5. The data used for the development and testing of the model are in Additional file 6, Additional file 7 and Additional file 8 which correspond to training, internal test and external validation set, respectively. The data in the files are normalized and can be thus directly used to obtain predictions using the model. After selecting and importing the training set (using the button “Select file with objects”) and the model (using the button “Select CPANN model”) the predictions for the training set can be made using the button “Make predictions for the objects”. After the predictions are obtained, the checkbox “Save existing dataset data” is selected to save the results for later use by the software. The same can be done for the other two sets. When the predictions for all the sets are obtained the button “Draw results” should be pressed to open the interface shown in Fig. 9. The interface can now be used as mentioned in the previous section. The interface in Fig. 9 shows a part of the top-map which was obtained using the data for acute toxicity. To show 2D structures of the compounds “Show structures” checkbox was selected and the smiles from Additional file 9 were imported. Figure 10a shows the neuron which was excited by external set object with ID = 7 which will be used here for demonstration purposes. The same neuron is visible also on the top-map shown in Fig. 9. To show the neuron on Fig. 10a, the user should select 7 from drop-down list available under “Select object ID” and then press the button “Find selected object”. After the button is pressed, a window showing the neuron will appear and the visible area of the top map will change so that the region of the top-map with the neuron will be visible. Figure 10b shows each of the compounds in its own window with the information regarding the compound.

The predicted value of −log(LC50) [log—common logarithm, LC50—concentration of the compounds which kills 50% of organisms (rainbow trout in our case)] for all the compounds was 1.99 (“pred.1” indicates prediction for the first target), which in this case matches the arithmetic mean of −log(LC50) values of two training set compounds that excited the neuron. The compound with ID = 7 is the only compound from external set that excited this neuron, therefore we may use other three compounds for read-across. When we look at the experimental values (“exp.1” indicates experimental value for the first target) of the compounds we can observe that the values are not the same. The bottom-left compound (ID = 145) has the highest experimental values 2.64, the upper-right compound (ID = 126) which has one methyl group less has experimental value 2.19, and the compound with two chlorines (ID = 26) has experimental value 1.79. For read-across, we selected compound 126 as the most similar to compound 7. Thus, we may say that −log(LC50) value predicted by read-across for the compound 7 is 2.19. Further, it can be observed that −log(LC50) value is smaller on the compound with one methyl group than on the compound with two methyl groups. Thus we could expect lower experimental value for the compound 7. The actual experimental value for compound 7 is 1.83. If we knew in this particular case that a linear relationship exists, we could use the compounds 126 and 145 for the linear regression where −log(LC50) depends on the number of methyl groups. The calculated linear regression would predict 1.74 for −log(LC50) value for a compound without any methyl group.

An inspection of the whole top-map shows that the compounds in the dataset used are structurally very different which makes the read-across method difficult to apply. From 69 compounds in the external set we could perform read-across for only 24 compounds. In the case of compound 7, the read-across value was slightly higher from the predicted one. However, when we performed some analysis of RMSE of –log(LC50) values predicted by the model and by read-across for the 24 compounds, we observed that RMSE was lower for read-across predictions. The highest error in read-across was made for acetaldehyde (ID = 80) based on acetone (ID = 74) data. In this case, the model made larger error. When the predictions for the acetaldehyde were not considered, the RMSE error calculated from predictions for 23 compounds was 0.80 for the model predictions and 0.49 for the read-across predictions. Among 24 read-across predictions, 18 predictions were made using a compound that excited the same neuron as the compound under consideration. The results of read-across predictions are included within the article in Additional file 10.

In the second example, the model will be built using bio-concentration factor (BCF) data obtained from the article written by Gissi et al. [13]. The descriptors used were the same as those reported in the supplementary material of the article for MLR method with 10 descriptors. Descriptors were calculated using Dragon 7.0 software for molecular descriptor calculation [14]. The data used can be found in supplementary material. The Additional file 11, Additional file 12 and Additional file 13 contain training set, internal validation (test) set and blind (external) set data, respectively. The smiles of the structures are available in the Additional file 15.

In this example, the number of neurons used will be small in comparison to the number of objects used in the training set. This will cause that the top-map will be densely populated while similar compounds will still be grouped together and will excite the same or similar neurons. In the input files “tab” is used as a delimiter, therefore the item “tab delimited” should be selected in the main window from the list used to define the delimiter for the file with object data. Training set should be imported as the first set. Then a check box “Use as normalization set” should be selected and the button “Normalize current descriptor data” should be pressed. In this way, the normalization factors are calculated from the training set data and the training set is normalized. Using these normalized data a new model can be built using “Train CPANN” button. The training parameters required and their values for this example (in brackets) are random seed (1234), number of neurons in x direction (9), number of neurons in y direction (9), toroid boundary conditions (Non-toroid NN), type of neighbourhood correction (Triangular), furthest neuron for correction (9), maximal learning rate (0.47), minimal learning rate (0.04), type of the best match (neuron with the weights most similar to the input) and number of epochs (161). The same parameters can also be found in Fig. 5 which shows a dialog box that is used to enter CPANN training parameters. The resulting model will be saved in file “*modelweights.unw*”. For this example, the resulting model file is given as Additional file 14. After the training, we can perform model validation by pressing the button “Model validation”. Then the predictions can be made and dataset data can be saved for further use by the software. After importing each of the other sets, the normalization of descriptor data should be done using training set data and then predictions can be made.

When the predictions are obtained for all the sets, a CPANN top-map can be shown. Additional file 15 should be selected when asked for the file with IDs and smiles. Using the model, we tried to perform read-across for the structures in the blind set. For approximately half of the compounds in the blind set we made read-across. The RMSE of 37 model predictions was 0.79, while the RMSE of read-across predictions for the same compounds was 0.55. Among 37 read-across predictions 30 predictions were made using a compound that excited the same neuron as the compound under consideration. The results of read-across predictions are included within the article in Additional file 16.

**Fig. 11:**
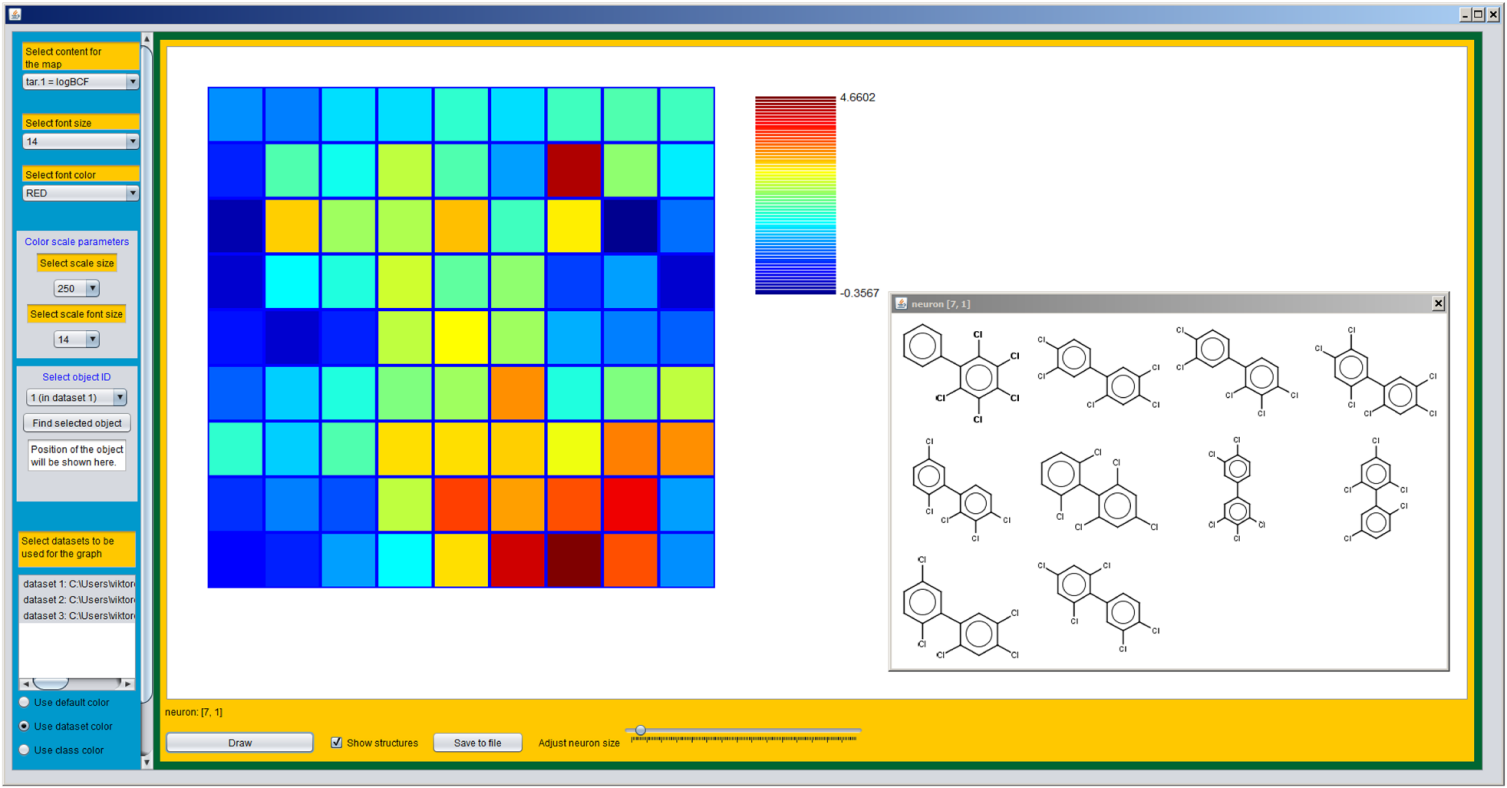
Response surface of the model and the neuron corresponding to the highest response value. The highest response value is at position [1, 7]. The structures that excited the neuron are polychlorinated biphenyls with log(BCF) above 4

The interface can be used also to identify neurons which have for example large or small weight value for certain descriptor or response. Subsequently, compounds with similar descriptor or target values can be identified. For example, if we wish to identify compounds with high log(BCF) then we first draw response by selecting “tar.1 = logBCF” from drop-down menu on the blue panel an redraw the map. The neuron with the highest response can be found based on the available colour scale. In the same way as before we can now display the neuron in a new window and identify the structures which excite the neuron. As can be found from the response surface, the compounds which have highest log(BCF) and are of the highest concern in this dataset are polychlorinated biphenyls which are commonly abbreviated as PCBs. The response surface and the neuron corresponding to the highest response are shown in Fig. 11.

**Fig. 12:**
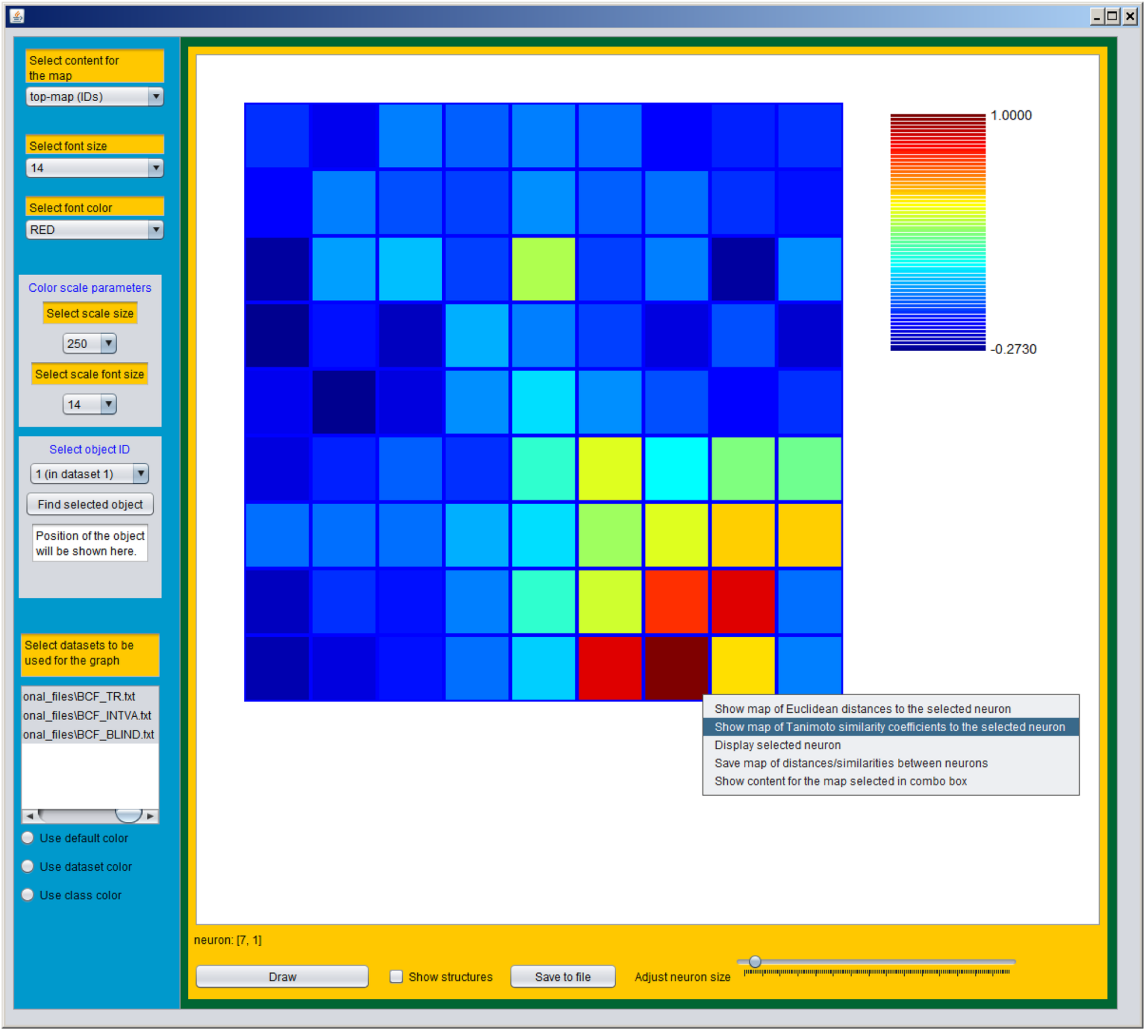
Surface plot of Tanimoto similarity coefficients for the neuron with the highest response value

Similarity between the selected neuron and other neurons on the map can be evaluated using Tanimoto similarity coefficient or Euclidean distance between the neurons. This can be done using a right-click on the neuron and selecting the preferred similarity measure from the pop-up menu. An example of the resulting surface plot corresponding to the selected similarity measure is given in Fig. 12. The second item “Show map of Tanimoto similarity coefficients to the selected neuron” was selected from the pop-up menu, as shown in Fig. 12. The same neuron as before (i.e. the neuron with the highest response at the position [1, 7]) was selected to calculate Tanimoto similarity coefficients to all other neurons. In Fig. 12, the most similar neurons to the selected neuron are shown in red colours which correspond to relatively high values of Tanimoto coefficients.

The two examples shown above were described in detail. Some additional tests were also performed using other datasets. For that purpose the Sutherland’s eight datasets [15] were used and QuBiLS-MIDAS 3D-indices provided in the paper by García-Jacas et al. [16] were used to build CPANN models for the datasets. The eight datasets included datasets for angiotensin converting enzyme inhibitors (ACE), acetylcholinesterase inhibitors (ACHE), ligands for the benzodiazepine receptor (BZR), cyclooxygenase-2 inhibitors (COX2), dihydrofolate reductase inhibitors (DHFR), glycogen phosphorylase b inhibitors (GPB), thermolysin inhibitors (THER), and thrombin inhibitors (THR). The same splitting of the data into training and external set was used as in the previous publications. The models were evaluated by repeated leave-many-out cross-validation and Y-scrambling. Y-scrambling validation was decisive for the selection of the models’ size since correlation coefficient became higher when larger number of neurons was used in the model. The results obtained for the eight models and their use in read-across are available in Additional file 17. The models found did not show very good performance for external set objects. One of the possible reasons could be the splitting of the objects. It was found also that maximal and/or minimal values for the set under consideration were in most cases not included in the training set. Using the developed models, read-across was performed for external set objects and comparison was made between the model predictions and read-across predictions for the objects where read-across could be performed. For six datasets read-across showed better prediction performance, and for two datasets better prediction performance was obtained using model predictions.

### Comparison with the Kohonen and CP-ANN toolbox

The software described within this paper is not the only one existing for development of CPANN models; nevertheless it offers unique possibilities for effective read-across on training/test data. The Kohonen and CP-ANN toolbox with similar functionality was recently developed in Milano Chemometrics and QSAR Research Group [17]. The software was developed as a toolbox to be used in Matlab. The learning algorithm used in the toolbox is essentially based on the same algorithm for Kohonen and counter-propagation artificial neural networks [18] as in this manuscript. One of the valuable properties of the toolbox is that its methods can be directly used through command prompt in Matlab apart of the provided GUI. This gives the user the possibility to use the methods in new Matlab applications. For the preparation of the data, the toolbox range scales the data and offers some additional options for data scaling. On the other hand, CPANNatNIC software accepts the data “as is” or offers standardization of independent variables based on the training set data. Both applications provide model weights and the possibility to visualize the results. The toolbox additionally gives the user an opportunity to analyse the weights of the model by using principal component analysis (PCA) to investigate the relationship between the variables used in the model. Such PCA analysis is not available in CPANNatNIC software. While both applications provide similar visualization of the results, CPANNatNIC software has different visualisation features and can also visualize 2D chemical structures from SMILES on the Kohonen map to help in the interpretation of the results and to facilitate read-across. Additionally, CPANNatNIC provides an option for locating an object on a top-map which may be needed when there are many objects on the top-map or the map has a large number of neurons. While the Kohonen and CP-ANN toolbox and CPANNatNIC are both freely available, the Matlab toolbox requires access to Matlab which is not freely available and CPANNatNIC requires freely available Java environment and CDK library.

## Conclusions

We present a program for building counter-propagation neural network models with an interface for viewing top-maps, descriptor levels and response surface. 2D representations of compounds can be shown on the top-map. This is useful when performing read-across for identification of similar compounds. The program provides simple interface which can be used to quickly find neuron excited by the compound under consideration. Thus, similar structures can be quickly identified and also used for read-across. Since the user both provides the dataset for the modelling and can develop new models, the model predictions as well as read-across predictions are not limited to any specific endpoint.

CPANNatNIC will be further developed in the future. We are planning to add features, such as descriptor selection and optimization, which will simplify model development process. Also, the representation of the objects within the software will be modified so that new information regarding the objects can be added and displayed within the software.

## Additional files

**Additional file 1.** File containing CPANNatNIC program.

**Additional file 2.** File containing CPANNatNIC source files.

**Additional file 3.** Example input data file.

**Additional file 4.** Example Excel file used to prepare file Example_input_prepared_in_Excel.txt.

**Additional file 5.** The model file for acute toxicity.

**Additional file 6.** Training set input file for acute toxicity.

**Additional file 7.** Test set input file for acute toxicity.

**Additional file 8.** Validation set input file for acute toxicity.

**Additional file 9.** The file containing smiles of compounds used for modelling acute toxicity.

**Additional file 10.** File with read-across results for acute toxicity validation set.

**Additional file 11.** Training set input file used for the modelling of bio-concentration factor.

**Additional file 12.** Internal test set input file used for the modelling of bio-concentration factor.

**Additional file 13.** External/blind set input file used for the modelling of bio-concentration factor.

**Additional file 14.** The resulting model for bio-concentration factor.

**Additional file 15.** The file containing smiles of compounds used for modelling bio-concentration factor.

**Additional file 16.** File with read-across results for bio-concentration factor external set.

**Additional file 17.** File containing results obtained for additional tests on eight datasets.
