# Supplementary material for: CPANNatNIC software for counter-propagation neural network to assist in read-across
Source: J Cheminform. 2017 May 22;9:30. doi: 10.1186/s13321-017-0218-y (PMC5440416; doi:10.1186/s13321-017-0218-y)
Supplement: Supplementary file 10 — Additional file 10. File with read-across results for acute toxicity validation set. [file 13321_2017_218_MOESM10_ESM.docx]

**Read-across results for prediction of acute toxicity (−log(LC50)) to rainbow trout.**

| **No** | **Compound’s ID**  (Validation set - RT) | **Position**  (neuron) | **Euclidean distance**  **to the neuron** | **The most similar object**  (exp. value) | **Euclidean distance**  **to the neuron** | **Compound’s experimental value** | **Predicted value by**  CP-ANN model* | **READ -ACROSS** |
| --- | --- | --- | --- | --- | --- | --- | --- | --- |
| 1 | 7 | [17,6] | 2.99 | **126**  (2.19) | 1.91 | 1.83 | 1.99 | **2.19** |
| 2 | 14 | [18,4] | 4.01 | **101**  (1.96) | 3.10 | 1.28 | 1.34 | **1.96** |
| 3 | 25 | [18,7] | 5.85 | **26**  (1.79) | 1.96 | 1.91 | 1.77 | **1.79** |
| 4 | 40 | [16,12] | 3.45 | **340**  (1.54) | 0.002 | 0.76 | 2.69 | **1.54** |
| 5 | 57 | [7,16] | 0.80 | **21**  (3.55) | 1.47 | 2.53 | 3.20 | **3.55** |
| 6 | 75 | [14,1] | 7.25 | **79**  (0.41) | 5.65 | 0.68 | 1.12 | **0.41** |
| 7 | 80 | [11,1] | 4.27 | **74**  (-1.87) | 2.08 | 1.30 | -2.31 | **-1.87** |
| 8 | 87 | [8,10] | 4.50 | **118**  (0.54) | 3.08 | 1.27 | 1.50 | **0.54** |
| 9 | 95 | [18,2] | 4.16 | **97**  (1.99) | 4.16 | 1.62 | 1.51 | **1.99** |
| **No** | **Compound’s ID**  (Validation set - RT) | **Position**  (neuron) | **Euclidean distance**  **to the neuron** | **The most similar object**  (exp. value) |  | **Compound’s experimental value** | **Predicted value by**  CP-ANN model* | **READ -ACROSS** |
| 10 | 100 | [19,12] | 2.67 | **99**  (0.34) | 1.68 | 0.05 | 1.07 | **0.34** |
| 11 | 111 | [17,1] | 0.94 | **12**  (1.10) | 0.004 | 1.13 | 1.10 | **1.10** |
| 12 | 138 | [1,13] | 1.51 | **139**  (2.19) | 1.37 | 2.55 | 2.59 | **2.19** |
| 13 | 158 | [8,13] | 3.26 | **44**  (3.61) | 0.005 | 4.08 | 3.61 | **3.61** |
| 14 | 168 | [11,18] | 2.53 | **190**  (2.08) | 0.004 | 3.17 | 2.08 | **2.08** |
| 15 | 215 | [17,7] | 5.57 | **248**  (3.89) | 0.004 | 4.21 | 3.89 | **3.89** |
| 16 | 219 | [4,12] | 3.52 | **220**  (0.05) | 0.004 | 0.01 | 1.71 | **0.05** |
| 17 | 221 | [4,12] | 3.29 | **220**  (0.05) | 0.004 | -0.08 | 1.71 | **0.05** |
| 18 | 223 | [9,13] | 2.18 | **160**  (2.90) | 2.87 | 2.59 | 3.31 | **2.90** |
| 19 | 236 | [17,12] | 4.73 | **198**  (2.09) | 0.003 | 2.09 | 2.09 | **2.09** |
| **No** | **Compound’s ID**  (Validation set - RT) | **Position**  (neuron) | **Euclidean distance**  **to the neuron** | **The most similar object**  (exp. value) |  | **Compound’s experimental value** | **Predicted value by**  CP-ANN model* | **READ -ACROSS** |
| 20 | 247 | [2,10] | 2.22 | **286**  (1.29) | 2.17 | 1.02 | 1.74 | **1.29** |
| 21 | 249 | [11,6] | 1.60 | **195**  (0.98) | 1.02 | 1.44 | 1.37 | **0.98** |
| 22 | 294 | [1,13] | 3.51 | **140**  (2.84) | 2.53 | 2.58 | 2.59 | **2.84** |
| 23 | 298 | [1,16] | 2.77 | **196**  (3.95) | 2.97 | 3.62 | 2.99 | **3.95** |
| 24 | 345 | [2,10] | 3.00 | **286**  (1.29) | 2.17 | 1.84 | 1.74 | **1.29** |
