# Supplementary material for: CPANNatNIC software for counter-propagation neural network to assist in read-across
Source: J Cheminform. 2017 May 22;9:30. doi: 10.1186/s13321-017-0218-y (PMC5440416; doi:10.1186/s13321-017-0218-y)
Supplement: Supplementary file 16 — Additional file 16. File with read-across results for bio-concentration factor external set. [file 13321_2017_218_MOESM16_ESM.docx]

**Read-across results for prediction of log(BCF).**

| **No** | **Compound’s ID**  (Validation set - RT) | **Position**  (neuron) | **Euclidean distance**  **to the neuron** | **The most similar object**  (exp. value) | **Euclidean distance**  **to the neuron** | **Compound’s experimental value** | **Predicted value by**  CP-ANN model* | **READ -ACROSS** |
| --- | --- | --- | --- | --- | --- | --- | --- | --- |
| 1 | 43 | [3,1] | 0.35 | **42**  (1.90) | 0.45 | 1.62 | 1.06 | **1.90** |
| 2 | 53 | [3,1] | 0.35 | **56**  (1.03) | 0.46 | 0.53 | 1.06 | **1.03** |
| 3 | 69 | [1,8] | 1.13 | **102**  (0.46) | 1.21 | 0.9 | 0.40 | **0.46** |
| 4 | 74 | [1,4] | 0.75 | **64**  (0.28) | 0.73 | 0.78 | 0.74 | **0.28** |
| 5 | 107 | [1,2] | 0.33 | **362**  (0.40) | 0.33 | 0.40 | 0.47 | **0.40** |
| 6 | 111 | [5,1] | 0.36 | **112**  (3.63) | 0.56 | 3.11 | 2.91 | **3.63** |
| 7 | 155 | [1,2] | 0.53 | **161**  (0.91) | 0.62 | 0.64 | 0.47 | **0.91** |
| 8 | 182 | [2,2] | 0.17 | **170**  (0.66) | 0.29 | 0.99 | 0.89 | **0.66** |
| 9 | 189 | [4,2] | 0.52 | **207**  (1.36) | 0.57 | 1.84 | 2.50 | **1.36** |
| 10 | 190 | [6,3] | 0.60 | **329**  (2.54) | 0.60 | 2.67 | 3.00 | **2.54** |
| **No** | **Compound’s ID**  (Validation set - RT) | **Position**  (neuron) | **Euclidean distance**  **to the neuron** | **The most similar object**  (exp. value) | **Euclidean distance**  **to the neuron** | **Compound’s experimental value** | **Predicted value by**  CP-ANN model* | **READ -ACROSS** |
| 11 | 205 | [4,2] | 0.57 | **207**  (1.36) | 0.57 | 1.43 | 2.50 | **1.36** |
| 12 | 213 | [6,9] | 1.30 | **465**  (1.48) | 1.19 | 0.32 | 1.38 | **1.48** |
| 13 | 223 | [5,5] | 0.34 | **224**  (3.4) | 0.24 | 2.81 | 2.76 | **3.4** |
| 14 | 238 | [8,5] | 0.77 | **429**  (1.03) | 0.76 | 0.96 | 0.90 | **1.03** |
| 15 | 296 | [5,3] | 1.08 | **7**  (2.8) | 1.20 | 2.03 | 2.93 | **2.80** |
| 16 | 305 | [9,4] | 0.90 | **783**  (2.05) | 1.00 | 1.29 | 2.45 | **2.05** |
| 17 | 318 | [2,1] | 0.18 | **364**  (0.36) | 0.38 | 0.2 | 0.43 | **0.36** |
| 18 | 320 | [1,2] | 0.74 | **311** (-0.19) | 0.61 | 1.02 | 0.47 | **-0.19** |
| 19 | 332 | [5,5] | 1.67 | **365**  (1.38) | 1.49 | 1.65 | 2.76 | **1.38** |
| 20 | 339 | [7,5] | 0.47 | **132**  (1.81) | 0.47 | 1.32 | 1.10 | **1.81** |
| **No** | **Compound’s ID**  (Validation set - RT) | **Position**  (neuron) | **Euclidean distance**  **to the neuron** | **The most similar object**  (exp. value) | **Euclidean distance**  **to the neuron** | **Compound’s experimental value** | **Predicted value by**  CP-ANN model* | **READ -ACROSS** |
| 21 | 363 | [6,3] | 0.88 | **202**  (3.31) | 0.40 | 3.54 | 3.00 | **3.31** |
| 22 | 368 | [7,2] | 0.59 | **366**  (3.66) | 0.59 | 3.12 | 3.69 | **3.66** |
| 23 | 377 | [6,5] | 0.92 | **269**  (3.20) | 0.69 | 4.18 | 2.32 | **3.20** |
| 24 | 392 | [7,4] | 0.96 | **14**  (2.01) | 0.96 | 2.15 | 1.67 | **2.01** |
| 25 | 445 | [1,3] | 0.66 | **765**  (1.64) | 0.48 | 0.68 | 1.72 | **1.64** |
| 26 | 458 | [5,1] | 0.11 | **542**  (3.81) | 0.12 | 3.81 | 2.91 | **3.81** |
| 27 | 467 | [6,5] | 0.75 | **269**  (3.20) | 0.69 | 3.59 | 2.32 | **3.20** |
| 28 | 496 | [6,2] | 0.32 | **430**  (2.63) | 0.32 | 3.53 | 3.27 | **2.63** |
| 29 | 552 | [6,1] | 0.22 | **551**  (4.39) | 0.20 | 4.71 | 4.32 | **4.39** |
| 30 | 597 | [7,7] | 1.10 | **152**  (0.34) | 1.32 | 0.35 | 2.84 | **0.34** |
| 31 | 609 | [8,1] | 1.40 | **366**  (3.66) | 0.59 | 3.18 | 3.67 | **3.66** |
| **No** | **Compound’s ID**  (Validation set - RT) | **Position**  (neuron) | **Euclidean distance**  **to the neuron** | **The most similar object**  (exp. value) | **Euclidean distance**  **to the neuron** | **Compound’s experimental value** | **Predicted value by**  CP-ANN model* | **READ -ACROSS** |
| 32 | 651 | [5,6] | 0.43 | **630**  (2.00) | 0.53 | 2.11 | 1.99 | **2.00** |
| 33 | 664 | [3,7] | 1.11 | **663**  (2.20) | 1.11 | 2.22 | 2.35 | **2.20** |
| 34 | 682 | [7,4] | 0.84 | **843**  (0.84) | 0.77 | 1.46 | 1.67 | **0.84** |
| 35 | 742 | [4,7] | 0.46 | **779**  (2.70) | 0.96 | 2.55 | 2.38 | **2.70** |
| 36 | 821 | [1,7] | 1.66 | **806**  (0.11) | 1.20 | -0.89 | -0.19 | **0.11** |
| 37 | 837 | [7,4] | 1.24 | **839**  (2.17) | 1.24 | 2.51 | 1.67 | **2.17** |
