# Supplementary material for: CPANNatNIC software for counter-propagation neural network to assist in read-across
Source: J Cheminform. 2017 May 22;9:30. doi: 10.1186/s13321-017-0218-y (PMC5440416; doi:10.1186/s13321-017-0218-y)
Supplement: Supplementary file 17 — Additional file 17. File containing results obtained for additional tests on eight datasets. [file 13321_2017_218_MOESM17_ESM.zip › ache/ACHE_read-across_results.docx]

**Read-across results for ACHE external set**

| **No** | **Compound’s ID** | **Position**  (neuron) | **Euclidean distance**  **to the neuron** | **The most similar object**  (exp. value) | **Euclidean distance**  **to the neuron** | **Compound’s experimental value** | **Predicted value by**  CP-ANN model* | **READ -ACROSS** |
| --- | --- | --- | --- | --- | --- | --- | --- | --- |
| 1 | 75 | [3,4] | 2.76 | 3  (5.52) | 1.84 | 4.57 | 4.86 | **5.52** |
| 2 | 76 | [1,1] | 1.18 | 12  (7.96) | 1.30 | 8.06 | 8.04 | **7.96** |
| 3 | 77 | [1,1] | 2.62 | 9  (8.92) | 2.03 | 8.66 | 8.04 | **8.92** |
| 4 | 79 | [2,2] | 1.57 | 4  (7.90) | 1.06 | 8.05 | 7.36 | **7.90** |
| 5 | 80 | [1,1] | 2.29 | 9  (8.92) | 2.03 | 6.47 | 8.04 | **8.92** |
| 6 | 83 | [1,1] | 2.02 | 2  (7.52) | 1.04 | 7.64 | 8.04 | **7.52** |
| 7 | 84 | [2,2] | 1.26 | 16  (6.10) | 0.73 | 5.92 | 8.04 | **6.10** |
| 8 | 87 | [2,2] | 1.93 | 19  (6.23) | 1.30 | 7.19 | 7.36 | **6.23** |
| 9 | 88 | [3,2] | 0.73 | 26  (6.69) | 0.83 | 6.15 | 6.89 | **6.69** |
| 10 | 89 | [3,2] | 1.16 | 25  (7.34) | 1.07 | 7.19 | 6.89 | **7.34** |
| **No** | **Compound’s ID** | **Position**  (neuron) | **Euclidean distance**  **to the neuron** | **The most similar object**  (exp. value) | **Euclidean distance**  **to the neuron** | **Compound’s experimental value** | **Predicted value by**  CP-ANN model* | **READ -ACROSS** |
| 11 | 92 | [1,2] | 1.84 | 34  (9.52) | 0.66 | 9.22 | 8.72 | **9.52** |
| 12 | 93 | [4,3] | 1.54 | 39  (4.85) | 1.42 | 6.43 | 6.16 | **4.85** |
| 13 | 94 | [1,3] | 2.03 | 1  (6.25) | 0.97 | 4.89 | 6.81 | **6.25** |
| 14 | 98 | [3,2] | 1.95 | 46  (6.64) | 1.46 | 6.06 | 6.89 | **6.64** |
| 15 | 99 | [2,2] | 1.26 | 50  (7.07) | 1.03 | 7.06 | 7.36 | **7.07** |
| 16 | 100 | [3,2] | 2.31 | 81  (6.10) | 1.53 | 7.16 | 6.89 | **6.10** |
| 17 | 105 | [1,3] | 1.08 | 59  (8.19) | 0.87 | 7.09 | 6.81 | **8.19** |
| 18 | 110 | [2,1] | 2.83 | 66  (7.52) | 2.58 | 6.52 | 7.48 | **7.52** |
