# Supplementary material for: CPANNatNIC software for counter-propagation neural network to assist in read-across
Source: J Cheminform. 2017 May 22;9:30. doi: 10.1186/s13321-017-0218-y (PMC5440416; doi:10.1186/s13321-017-0218-y)
Supplement: Supplementary file 17 — Additional file 17. File containing results obtained for additional tests on eight datasets. [file 13321_2017_218_MOESM17_ESM.zip › bzr/BZR_read-across_results.docx]

**Read-across results for BZR external set**

| **No** | **Compound’s ID** | **Position**  (neuron) | **Euclidean distance**  **to the neuron** | **The most similar object**  (exp. value) | **Euclidean distance**  **to the neuron** | **Compound’s experimental value** | **Predicted value by**  CP-ANN model* | **READ -ACROSS** |
| --- | --- | --- | --- | --- | --- | --- | --- | --- |
| 1 | 101 | [2,4] | 1.01 | 9  (8.42) | 1.00 | 8.15 | 8.65 | **8.42** |
| 2 | 102 | [2,4] | 1.17 | 16  (8.03) | 1.06 | 8.00 | 8.65 | **8.03** |
| 3 | 104 | [2,4] | 1.70 | 13  (8.13) | 0.49 | 7.74 | 8.65 | **8.13** |
| 4 | 105 | [2,1] | 1.45 | 11  (8.29) | 1.32 | 7.52 | 7.26 | **8.29** |
| 5 | 108 | [1,4] | 3.46 | 22  (7.74) | 2.06 | 7.23 | 7.78 | **7.74** |
| 6 | 109 | [3,1] | 2.42 | 29  (7.47) | 2.82 | 7.06 | 7.59 | **7.47** |
| 7 | 110 | [2,1] | 1.76 | 116  (6.21) | 2.95 | 6.96 | 7.26 | **6.21** |
| 8 | 112 | [2,1] | 1.46 | 41  (6.82) | 1.02 | 6.52 | 7.26 | **6.82** |
| 9 | 115 | [2,2] | 1.35 | 40  (7.02) | 1.12 | 6.34 | 7.46 | **7.02** |
| 10 | 116 | [2,1] | 2.95 | 110  (6.96) | 1.76 | 6.21 | 7.26 | **6.96** |
| **No** | **Compound’s ID** | **Position**  (neuron) | **Euclidean distance**  **to the neuron** | **The most similar object**  (exp. value) | **Euclidean distance**  **to the neuron** | **Compound’s experimental value** | **Predicted value by**  CP-ANN model* | **READ -ACROSS** |
| 11 | 118 | [4,4] | 1.17 | 59  (8.32) | 1.42 | 8.48 | 8.03 | **8.32** |
| 12 | 119 | [4,4] | 1.46 | 74  (7.96) | 1.60 | 8.38 | 8.03 | **7.96** |
| 13 | 120 | [4,3] | 1.72 | 55  (8.54) | 1.81 | 8.85 | 8.46 | **8.54** |
| 14 | 121 | [4,4] | 1.44 | 60  (8.13) | 1.09 | 8.35 | 8.03 | **8.13** |
| 15 | 123 | [4,3] | 2.18 | 70  (8.38) | 1.80 | 7.85 | 8.46 | **8.38** |
| 16 | 126 | [4,4] | 1.95 | 64  (8.19) | 1.24 | 8.30 | 8.03 | **8.19** |
| 17 | 129 | [4,4] | 1.93 | 64  (8.19) | 1.24 | 7.61 | 8.03 | **8.19** |
| 18 | 130 | [1,2] | 3.14 | 68  (7.15) | 1.62 | 8.41 | 6.94 | **7.15** |
| 19 | 131 | [4,3] | 1.68 | 69  (8.60) | 1.58 | 8.82 | 8.46 | **8.60** |
| 20 | 132 | [3,4] | 2.49 | 71  (7.80) | 1.78 | 7.82 | 7.77 | **7.80** |
| 21 | 135 | [3,4] | 2.67 | 58  (8.46) | 1.78 | 8.55 | 7.77 | **8.46** |
| **No** | **Compound’s ID** | **Position**  (neuron) | **Euclidean distance**  **to the neuron** | **The most similar object**  (exp. value) | **Euclidean distance**  **to the neuron** | **Compound’s experimental value** | **Predicted value by**  CP-ANN model* | **READ -ACROSS** |
| 22 | 136 | [3,4] | 1.77 | 76  (7.96) | 1.31 | 8.72 | 7.77 | **7.96** |
| 23 | 137 | [4,2] | 1.38 | 89  (8.21) | 0.69 | 8.77 | 8.13 | **8.21** |
| 24 | 138 | [4,1] | 1.20 | 79  (8.64) | 0.89 | 8.77 | 8.46 | **8.64** |
| 25 | 139 | [4,1] | 4.38 | 85  (8.48) | 1.41 | 8.55 | 8.46 | **8.48** |
| 26 | 141 | [3,2] | 1.72 | 96  (8.57) | 0.95 | 8.17 | 8.57 | **8.57** |
| 27 | 142 | [1,2] | 3.09 | 68  (7.15) | 1.62 | 8.03 | 6.94 | **7.15** |
