# Supplementary material for: CPANNatNIC software for counter-propagation neural network to assist in read-across
Source: J Cheminform. 2017 May 22;9:30. doi: 10.1186/s13321-017-0218-y (PMC5440416; doi:10.1186/s13321-017-0218-y)
Supplement: Supplementary file 17 — Additional file 17. File containing results obtained for additional tests on eight datasets. [file 13321_2017_218_MOESM17_ESM.zip › cox2/COX2_read-across_results.docx]

**Read-across results for COX2 external set**

| **No** | **Compound’s ID** | **Position**  (neuron) | **Euclidean distance**  **to the neuron** | **The most similar object**  (exp. value) | **Euclidean distance**  **to the neuron** | **Compound’s experimental value** | **Predicted value by**  CP-ANN model* | **READ -ACROSS** |
| --- | --- | --- | --- | --- | --- | --- | --- | --- |
| 1 | 191 | [7,4] | 2.35 | 1  (7.22) | 2.23 | 7.85 | 7.55 | **7.22** |
| 2 | 193 | [6,7] | 1.50 | 6  (6.92) | 1.39 | 5.49 | 7.57 | **6.92** |
| 3 | 195 | [3,6] | 2.46 | 4  (7.10) | 0.92 | 7.70 | 6.35 | **7.10** |
| 4 | 196 | [5,7] | 1.81 | 6  (6.92) | 1.39 | 6.33 | 7.50 | **6.92** |
| 5 | 197 | [3,3] | 1.47 | 9  (5.84) | 1.43 | 6.85 | 6.52 | **5.84** |
| 6 | 199 | [2,4] | 1.80 | 21  (6.46) | 0.42 | 6.24 | 6.48 | **6.46** |
| 7 | 202 | [2,5] | 1.21 | 47  (6.14) | 1.05 | 4.91 | 6.41 | **6.14** |
| 8 | 203 | [4,7] | 2.04 | 50  (5.80) | 0.61 | 5.24 | 6.41 | **5.80** |
| 9 | 204 | [2,7] | 3.93 | 53  (5.43) | 2.29 | 6.49 | 5.78 | **5.43** |
| 10 | 205 | [3,7] | 0.61 | 51  (6.64) | 0.72 | 5.81 | 6.51 | **6.64** |
| **No** | **Compound’s ID** | **Position**  (neuron) | **Euclidean distance**  **to the neuron** | **The most similar object**  (exp. value) | **Euclidean distance**  **to the neuron** | **Compound’s experimental value** | **Predicted value by**  CP-ANN model* | **READ -ACROSS** |
| 11 | 206 | [2,4] | 2.57 | 21  (6.46) | 0.42 | 4.17 | 6.48 | **6.46** |
| 12 | 208 | [1,1] | 1.45 | 60  (6.36) | 0.98 | 5.74 | 6.33 | **6.36** |
| 13 | 210 | [2,6] | 1.72 | 74  (6.20) | 0.86 | 5.96 | 6.24 | **6.20** |
| 14 | 212 | [2,5] | 0.49 | 75  (6.33) | 0.20 | 5.96 | 6.41 | **6.33** |
| 15 | 213 | [1,2] | 1.21 | 80  (6.96) | 1.29 | 6.37 | 7.41 | **6.96** |
| 16 | 216 | [1,4] | 0.39 | 75  (6.33) | 0.20 | 4.29 | 6.44 | **6.33** |
| 17 | 217 | [6,7] | 0.75 | 87  (7.12) | 1.23 | 7.28 | 7.57 | **7.12** |
| 18 | 218 | [4,7] | 2.00 | 82  (8.30) | 1.03 | 5.18 | 6.41 | **8.30** |
| 19 | 223 | [5,5] | 1.83 | 98  (6.82) | 1.27 | 6.77 | 8.05 | **6.82** |
| 20 | 226 | [6,6] | 1.63 | 100  (7.77) | 1.37 | 7.48 | 8.00 | **7.77** |
| 21 | 227 | [5,6] | 1,47 | 108  (7.88) | 0.58 | 7.88 | 7.83 | **7.88** |
| **No** | **Compound’s ID** | **Position**  (neuron) | **Euclidean distance**  **to the neuron** | **The most similar object**  (exp. value) | **Euclidean distance**  **to the neuron** | **Compound’s experimental value** | **Predicted value by**  CP-ANN model* | **READ -ACROSS** |
| 22 | 230 | [5,5] | 1.06 | 109  (8.30) | 0.88 | 8.30 | 8.05 | **8.30** |
| 23 | 231 | [5,5] | 1.06 | 111  (8.52) | 0.91 | 8.22 | 8.05 | **8.52** |
| 24 | 235 | [4,2] | 1.69 | 129  (6.37) | 1.09 | 6.19 | 7.05 | **6.37** |
| 25 | 236 | [5,1] | 1.34 | 126  (8.33) | 0.65 | 4.84 | 6.52 | **8.33** |
| 26 | 238 | [5,1] | 1.42 | 127  (4.95) | 1.04 | 4.33 | 6.52 | **4.95** |
| 27 | 249 | [5,2] | 0.94 | 145  (7.66) | 1.37 | 7.55 | 7.33 | **7.66** |
| 28 | 251 | [7,3] | 1.12 | 149  (6.12) | 1.55 | 6.12 | 7.42 | **6.12** |
| 29 | 253 | [4,1] | 3.07 | 120  (5.58) | 2.41 | 4.03 | 6.66 | **5.58** |
| 30 | 256 | [5,2] | 1.73 | 244  (5.96) | 2.03 | 4.15 | 7.33 | **5.96** |
| 31 | 265 | [7,3] | 1.38 | 130  (6.22) | 1.20 | 7.62 | 7.42 | **6.22** |
| 32 | 269 | [7.7] | 2.13 | 160  (9.00) | 1.32 | 8.52 | 7.99 | **9.00** |
| **No** | **Compound’s ID** | **Position**  (neuron) | **Euclidean distance**  **to the neuron** | **The most similar object**  (exp. value) | **Euclidean distance**  **to the neuron** | **Compound’s experimental value** | **Predicted value by**  CP-ANN model* | **READ -ACROSS** |
| 33 | 278 | [7,4] | 1.26 | 183  (7.24) | 1.28 | 7.10 | 7.55 | **7.24** |
