# Supplementary material for: CPANNatNIC software for counter-propagation neural network to assist in read-across
Source: J Cheminform. 2017 May 22;9:30. doi: 10.1186/s13321-017-0218-y (PMC5440416; doi:10.1186/s13321-017-0218-y)
Supplement: Supplementary file 17 — Additional file 17. File containing results obtained for additional tests on eight datasets. [file 13321_2017_218_MOESM17_ESM.zip › dhfr/DHFR_read-across_results.docx]

**Read-across results for DHFR external set**

| **No** | **Compound’s ID** | **Position**  (neuron) | **Euclidean distance**  **to the neuron** | **The most similar object**  (exp. value) | **Euclidean distance**  **to the neuron** | **Compound’s experimental value** | **Predicted value by**  CP-ANN model* | **READ -ACROSS** |
| --- | --- | --- | --- | --- | --- | --- | --- | --- |
| 1 | 238 | [4,4] | 1.45 | 2  (4.69) | 1.25 | 4.09 | 4.86 | **4.69** |
| 2 | 240 | [7,3] | 1.67 | 6  (6.96) | 0.62 | 7.42 | 6.11 | **6.96** |
| 3 | 242 | [2,2] | 1.16 | 4  (9.44) | 1.09 | 8.96 | 8.62 | **9.44** |
| 4 | 245 | [1,1] | 1.22 | 13  (4.99) | 0.39 | 4.44 | 4.57 | **4.99** |
| 5 | 246 | [1,2] | 2.51 | 17  (6.06) | 1.53 | 5.72 | 4.99 | **6.06** |
| 6 | 255 | [3,7] | 1.52 | 51  (7.77) | 1.97 | 7.16 | 6.54 | **7.77** |
| 7 | 256 | [2,7] | 1.09 | 38  (7.59) | 0.99 | 8.09 | 7.22 | **7.59** |
| 8 | 257 | [1,7] | 2.12 | 52  (6.30) | 1.84 | 7.59 | 6.79 | **6.30** |
| 9 | 259 | [1,7] | 2.74 | 31  (9.81) | 2.57 | 8.44 | 6.79 | **9.81** |
| 10 | 260 | [3,7] | 1.70 | 34  (7.14) | 1.69 | 7.64 | 6.54 | **7.14** |
| **No** | **Compound’s ID** | **Position**  (neuron) | **Euclidean distance**  **to the neuron** | **The most similar object**  (exp. value) | **Euclidean distance**  **to the neuron** | **Compound’s experimental value** | **Predicted value by**  CP-ANN model* | **READ -ACROSS** |
| 11 | 261 | [6,1] | 0.90 | 33  (8.23) | 1.02 | 8.01 | 7.79 | **8.23** |
| 12 | 263 | [6,1] | 1.44 | 32  (7.36) | 1.48 | 7.55 | 7.79 | **7.36** |
| 13 | 265 | [7,5] | 2.00 | 57  (4.38) | 0.72 | 5.10 | 6.08 | **4.38** |
| 14 | 266 | [2,1] | 1.37 | 59  (3.72) | 1.12 | 5.30 | 4.62 | **3.72** |
| 15 | 268 | [2,1] | 2.45 | 53  (5.92) | 2.60 | 6.27 | 4.62 | **5.92** |
| 16 | 272 | [1,5] | 2.27 | 63  (6.54) | 1.22 | 6.25 | 6.24 | **6.54** |
| 17 | 273 | [3,7] | 0.97 | 67  (6.82) | 0.96 | 7.47 | 6.54 | **6.82** |
| 18 | 275 | [2,7] | 2.36 | 64  (7.42) | 1.08 | 7.13 | 7.22 | **7.42** |
| 19 | 276 | [5,5] | 1.88 | 78  (6.77) | 1.89 | 7.10 | 6.87 | **6.77** |
| 20 | 279 | [4,2] | 1.12 | 94  (7.14) | 0.93 | 6.38 | 6.76 | **7.14** |
| 21 | 280 | [7,2] | 0,51 | 122  (7.24) | 0.44 | 7.33 | 7.45 | **7.24** |
| **No** | **Compound’s ID** | **Position**  (neuron) | **Euclidean distance**  **to the neuron** | **The most similar object**  (exp. value) | **Euclidean distance**  **to the neuron** | **Compound’s experimental value** | **Predicted value by**  CP-ANN model* | **READ -ACROSS** |
| 22 | 281 | [7,2] | 0.56 | 107  (7.82) | 1.49 | 8.29 | 7.45 | **7.82** |
| 23 | 282 | [6,2] | 2.00 | 109  (7.74) | 1.32 | 7.12 | 7.39 | **7.74** |
| 24 | 286 | [4,2] | 1.22 | 99  (7.80) | 1.17 | 8.57 | 6.76 | **7.80** |
| 25 | 288 | [5,1] | 2.04 | 129  (8.43) | 0.97 | 9.40 | 6.76 | **8.43** |
| 26 | 289 | [4,1] | 1.56 | 151  (6.09) | 1.09 | 5.85 | 6.04 | **6.09** |
| 27 | 290 | [6,5] | 1.00 | 159  (6.62) | 1.17 | 7.40 | 5.94 | **6.62** |
| 28 | 296 | [3,2] | 1.04 | 153  (7.48) | 1.00 | 7.52 | 5.99 | **7.48** |
| 29 | 302 | [7,7] | 2.09 | 143  (7.70) | 1.56 | 7.77 | 7.16 | **7.70** |
| 30 | 304 | [6,6] | 1.30 | 181  (7.53) | 1.22 | 6.64 | 7.37 | **7.53** |
| 31 | 307 | [7,6] | 1.47 | 169  (7.59) | 0.45 | 7.00 | 7.27 | **7.59** |
| 32 | 310 | [6,7] | 0.88 | 179  (6.39) | 0.75 | 7.36 | 6.44 | **6.39** |
| **No** | **Compound’s ID** | **Position**  (neuron) | **Euclidean distance**  **to the neuron** | **The most similar object**  (exp. value) | **Euclidean distance**  **to the neuron** | **Compound’s experimental value** | **Predicted value by**  CP-ANN model* | **READ -ACROSS** |
| 33 | 311 | [6,7] | 1.41 | 179  (6.39) | 0.75 | 5.52 | 6.44 | **6.39** |
| 34 | 312 | [7,6] | 1.65 | 135  (6.48) | 1.35 | 6.30 | 7.27 | **6.48** |
| 35 | 313 | [6,7] | 0.87 | 193  (6.77) | 0.82 | 6.82 | 6.44 | **6.77** |
| 36 | 321 | [2,6] | 1.87 | 196  (3.94) | 0.59 | 4.20 | 4.50 | **3.94** |
| 37 | 328 | [3,6] | 1.04 | 210  (6.40) | 1.24 | 6.43 | 5.29 | **6.40** |
| 38 | 329 | [3,4] | 0.64 | 211  (5.51) | 7e-4 | 5.23 | 5.51 | **5.51** |
| 39 | 338 | [3,5] | 1.54 | 225  (4.60) | 1.42 | 4.82 | 4.59 | **4.60** |
| 40 | 339 | [2,5] | 1.81 | 226  (4.06) | 1.01 | 4.25 | 4.43 | **4.06** |
| 41 | 340 | [1,4] | 0.95 | 232  (6.05) | 0.69 | 5.46 | 5.38 | **6.05** |
| 42 | 342 | [1,2] | 1.55 | 233  (4.96) | 1.54 | 3.60 | 4.99 | **4.96** |
| 43 | 348 | [6,3] | 1.84 | 10  (5.09) | 1.55 | 3.97 | 5.00 | **5.09** |
| **No** | **Compound’s ID** | **Position**  (neuron) | **Euclidean distance**  **to the neuron** | **The most similar object**  (exp. value) | **Euclidean distance**  **to the neuron** | **Compound’s experimental value** | **Predicted value by**  CP-ANN model* | **READ -ACROSS** |
| 44 | 351 | [7,7] | 1.61 | 192  (5.94) | 1.44 | 4.89 | 7.16 | **5.94** |
| 45 | 352 | [4,4] | 0.86 | 199  (4.14) | 0.89 | 4.45 | 4.86 | **4.14** |
| 46 | 354 | [2,6] | 0.85 | 203  (3.81) | 0.87 | 4.15 | 4.50 | **3.81** |
| 47 | 359 | [2,6] | 1.09 | 214  (4.77) | 0.64 | 4.48 | 4.50 | **4.77** |
| 48 | 361 | [5,4] | 1.96 | 334  (4.64) | 1.27 | 4.21 | 6.41 | **4.64** |
