# Supplementary material for: CPANNatNIC software for counter-propagation neural network to assist in read-across
Source: J Cheminform. 2017 May 22;9:30. doi: 10.1186/s13321-017-0218-y (PMC5440416; doi:10.1186/s13321-017-0218-y)
Supplement: Supplementary file 17 — Additional file 17. File containing results obtained for additional tests on eight datasets. [file 13321_2017_218_MOESM17_ESM.zip › gpb/GPB_read-across_results.docx]

**Read-across results for GPB external set**

| **No** | **Compound’s ID** | **Position**  (neuron) | **Euclidean distance**  **to the neuron** | **The most similar object**  (exp. value) | **Euclidean distance**  **to the neuron** | **Compound’s experimental value** | **Predicted value by**  CP-ANN model* | **READ -ACROSS** |
| --- | --- | --- | --- | --- | --- | --- | --- | --- |
| 1 | 45 | [2,2] | 1.45 | 6  (1.60) | 1.16 | 2.10 | 3.35 | **1.60** |
| 2 | 46 | [3,1] | 1.74 | 11  (2.40) | 1.73 | 2.30 | 2.30 | **2.40** |
| 3 | 47 | [1,2] | 1.07 | 12  (1.50) | 0.83 | 1.80 | 1.86 | **1.50** |
| 4 | 48 | [1,2] | 2.69 | 14  (2.10) | 3.80 | 2.00 | 1.86 | **2.10** |
| 5 | 52 | [2,1] | 2.26 | 25  (3.70) | 1.14 | 1.40 | 3.11 | **3.70** |
| 6 | 55 | [2,3] | 1.92 | 32  (3.40) | 0.78 | 3.90 | 3.74 | **3.40** |
| 7 | 60 | [2,1] | 1.93 | 52  (1.40) | 2.26 | 2.90 | 3.11 | **1.40** |
| 8 | 61 | [3,1] | 1.84 | 17  (2.60) | 1.80 | 2.60 | 2.30 | **2.60** |
| 9 | 63 | [2,1] | 2.26 | 35  (2.60) | 0.99 | 1.80 | 3.11 | **2.60** |
