# Supplementary material for: CPANNatNIC software for counter-propagation neural network to assist in read-across
Source: J Cheminform. 2017 May 22;9:30. doi: 10.1186/s13321-017-0218-y (PMC5440416; doi:10.1186/s13321-017-0218-y)
Supplement: Supplementary file 17 — Additional file 17. File containing results obtained for additional tests on eight datasets. [file 13321_2017_218_MOESM17_ESM.zip › ther/THER_read-across_results.docx]

**Read-across results for THER external set**

| **No** | **Compound’s ID** | **Position**  (neuron) | **Euclidean distance**  **to the neuron** | **The most similar object**  (exp. value) | **Euclidean distance**  **to the neuron** | **Compound’s experimental value** | **Predicted value by**  CP-ANN model* | **READ -ACROSS** |
| --- | --- | --- | --- | --- | --- | --- | --- | --- |
| 1 | 54 | [3,3] | 2.19 | 4  (2.51) | 1.73 | 2.54 | 3.33 | **2.51** |
| 2 | 61 | [2,1] | 1.24 | 15  (7.35) | 0.90 | 10.17 | 6.41 | **7.35** |
| 3 | 63 | [2,1] | 1.68 | 35  (6.17) | 1.68 | 4.38 | 6.41 | **6.17** |
| 4 | 66 | [1,2] | 1.41 | 6  (6.22) | 1.36 | 5.77 | 3.31 | **6.22** |
| 5 | 72 | [3,3] | 1.74 | 47  (5.59) | 0.93 | 5.16 | 3.33 | **5.59** |
| 6 | 75 | [2,2] | 1.55 | 14  (3.46) | 0.92 | 3.66 | 4.37 | **3.46** |
| 7 | 76 | [1,2] | 2.02 | 39  (5.57) | 2.14 | 3.42 | 3.31 | **5.57** |
