# Supplementary material for: CPANNatNIC software for counter-propagation neural network to assist in read-across
Source: J Cheminform. 2017 May 22;9:30. doi: 10.1186/s13321-017-0218-y (PMC5440416; doi:10.1186/s13321-017-0218-y)
Supplement: Supplementary file 17 — Additional file 17. File containing results obtained for additional tests on eight datasets. [file 13321_2017_218_MOESM17_ESM.zip › thr/THR_read-across_results.docx]

**Read-across results for THR external set**

| **No** | **Compound’s ID** | **Position**  (neuron) | **Euclidean distance**  **to the neuron** | **The most similar object**  (exp. value) | **Euclidean distance**  **to the neuron** | **Compound’s experimental value** | **Predicted value by**  CP-ANN model* | **READ -ACROSS** |
| --- | --- | --- | --- | --- | --- | --- | --- | --- |
| 1 | 60 | [2,1] | 3.95 | 46  (8.48) | 1.37 | 8.38 | 7.56 | **8.48** |
| 2 | 63 | [3,1] | 2.40 | 35  (6.16) | 2.36 | 7.75 | 6.34 | **6.16** |
| 3 | 64 | [2,2] | 2.05 | 6  (7.77) | 1.89 | 7.72 | 7.38 | **7.77** |
| 4 | 66 | [2,1] | 2.87 | 17  (7.05) | 2.92 | 7.50 | 7.56 | **7.05** |
| 5 | 67 | [1,2] | 2.74 | 27  (6.55) | 2.46 | 7.38 | 5.84 | **6.55** |
| 6 | 68 | [3,2] | 1.35 | 32  (6.30) | 0.83 | 7.38 | 6.94 | **6.30** |
| 7 | 70 | [1,3] | 3.12 | 11  (7.43) | 2.39 | 6.92 | 7.02 | **7.43** |
| 8 | 72 | [2,1] | 3.39 | 14  (7.23) | 1.64 | 6.75 | 7.56 | **7.23** |
| 9 | 73 | [3,3] | 2.32 | 21  (6.82) | 2.68 | 6.64 | 6.35 | **6.82** |
| 10 | 75 | [2,1] | 2.19 | 9  (7.59) | 1.36 | 6.46 | 7.56 | **7.59** |
| **No** | **Compound’s ID** | **Position**  (neuron) | **Euclidean distance**  **to the neuron** | **The most similar object**  (exp. value) | **Euclidean distance**  **to the neuron** | **Compound’s experimental value** | **Predicted value by**  CP-ANN model* | **READ -ACROSS** |
| 11 | 76 | [2,2] | 1.81 | 20  (6.82) | 1.97 | 6.29 | 7.38 | **6.82** |
| 12 | 78 | [3,3] | 2.37 | 37  (5.75) | 2.33 | 6.05 | 6.35 | **5.75** |
| 13 | 80 | [3,1] | 2.06 | 35  (6.16) | 2.36 | 5.68 | 6.34 | **6.16** |
| 14 | 81 | [3,2] | 2.10 | 24  (6.68) | 1.96 | 5.51 | 6.94 | **6.68** |
| 15 | 84 | [1,2] | 3.79 | 27  (6.55) | 2.46 | 4.52 | 5.84 | **6.55** |
| 16 | 86 | [1,2] | 3.10 | 44  (4.77) | 2.39 | 4.36 | 5.84 | **4.77** |
| 17 | 87 | [2,2] | 2.38 | 20  (6.82) | 1.97 | 6.59 | 7.38 | **6.82** |
