# Supplementary material for: CPANNatNIC software for counter-propagation neural network to assist in read-across
Source: J Cheminform. 2017 May 22;9:30. doi: 10.1186/s13321-017-0218-y (PMC5440416; doi:10.1186/s13321-017-0218-y)
Supplement: Supplementary file 17 — Additional file 17. File containing results obtained for additional tests on eight datasets. [file 13321_2017_218_MOESM17_ESM.zip › ace/ACE_read-across_results.docx]

**Read-across results for ACE external set**

| **No** | **Compound’s ID** | **Position**  (neuron) | **Euclidean distance**  **to the neuron** | **The most similar object**  (exp. value) | **Euclidean distance**  **to the neuron** | **Compound’s experimental value** | **Predicted value by**  CP-ANN model* | **READ -ACROSS** |
| --- | --- | --- | --- | --- | --- | --- | --- | --- |
| 1 | 77 | [4,2] | 4.46 | **2**  (9.0) | 0.87 | 6.15 | 5.03 | **9.0** |
| 2 | 83 | [4,4] | 1.55 | 3  (7.64) | 0.82 | 8.05 | 6.01 | **7.64** |
| 3 | 86 | [2,3] | 2.05 | 6  (8.54) | 0.50 | 9.64 | 7.94 | **8.54** |
| 4 | 88 | [2,2] | 0.66 | 14  (8.11) | 0.83 | 8.52 | 8.46 | **8.11** |
| 5 | 90 | [3,4] | 0.99 | 18  (6.07) | 0.76 | 5.8 | 4.8 | **6.07** |
| 6 | 91 | [1,3] | 1.54 | 20  (6.7) | 1.23 | 6.19 | 8.32 | **6.7** |
| 7 | 92 | [3,3] | 1.20 | 17  (5.55) | 0.95 | 2.7 | 3.62 | **5.55** |
| 8 | 94 | [2,4] | 2.16 | 20  (6.7) | 1.23 | 5.52 | 3.79 | **6.7** |
| 9 | 95 | [4,5] | 1.11 | 26  (2.98) | 0.84 | 3.21 | 5.03 | **2.98** |
| 10 | 96 | [4,2] | 1.18 | 29  (3.38) | 1.05 | 3.64 | 5.03 | **3.38** |
| **No** | **Compound’s ID** | **Position**  (neuron) | **Euclidean distance**  **to the neuron** | **The most similar object**  (exp. value) | **Euclidean distance**  **to the neuron** | **Compound’s experimental value** | **Predicted value by**  CP-ANN model* | **READ -ACROSS** |
| 11 | 97 | [3,3] | 1.05 | 33  (4.28) | 0.94 | 3.03 | 3.62 | **4.28** |
| 12 | 100 | [4,3] | 0.90 | 17  (5.55) | 0.95 | 5.62 | 3.53 | **5.55** |
| 13 | 102 | [3,4] | 1.21 | 19  (6.37) | 0.76 | 6.15 | 4.81 | **6.37** |
| 14 | 103 | [3,2] | 0.92 | 38  (5.31) | 1.11 | 4.99 | 7.53 | **5.31** |
| 15 | 104 | [4,1] | 1.14 | 40  (4.32) | 0.44 | 5.08 | 3.43 | **4.32** |
| 16 | 108 | [1,1] | 1.79 | 51  (9.88) | 0.71 | 6.47 | 8.99 | **9.88** |
| 17 | 109 | [1,3] | 0.81 | 53  (9.28) | 0.73 | 9.11 | 8.32 | **9.28** |
| 18 | 110 | [2,2] | 3.31 | 60  (8.97) | 1.00 | 9.54 | 8.46 | **8.97** |
| 19 | 112 | [4,4] | 1.25 | 70  (3.64) | 0.78 | 3.59 | 6.01 | **3.64** |
