# Supplementary material for: CPANNatNIC software for counter-propagation neural network to assist in read-across
Source: J Cheminform. 2017 May 22;9:30. doi: 10.1186/s13321-017-0218-y (PMC5440416; doi:10.1186/s13321-017-0218-y)
Supplement: Supplementary file 1 — Additional file 1. File containing CPANNatNIC program. [file 13321_2017_218_MOESM1_ESM.zip › CPANNatNIC.zip!/CPANNatNIC/User_guide_for_CPANNatNIC.docx]

**USER GUIDE FOR CPANNatNIC**

1. Introduction

This user manual gives explanation on how to prepare input data for the software and how to use the software for the modelling and visualization of the results. Only, the most relevant options of the software are described.

CPANNatNIC is software for building counter-propagation artificial neural network models. It can be used as a “SOM tool” to group object according to their similarity and was built with the intention to be used also as a “read-across tool” for chemical compounds. It provides simple graphical user interface for modelling and visualization of the modelling results.

2. Installation

To run CPANNatNIC, extract the ZIP archive to a folder. Make sure that “lib” folder is in the same folder as the program file CPANNatNIC.jar. You can try to run the program by clicking CPANNatNIC.jar. Otherwise, open command prompt and point it to the folder containing CPANNatNIC.jar. Then execute the following command from the command prompt: java -jar "CPANNatNIC.jar"

3. Data File format

The data in the input file should be formatted as it is described in this section.

The file extensions of the data file should be “txt” or “dat”. The delimiters used in the file can be space, comma or semicolon.

The first line of the file is reserved for short description of the file content.

The second line contains two numbers separated by a delimiter. The first number is the number of objects (=number of rows with dataset data) and the second number is the number of independent variables (descriptors) plus the number of endpoints (targets) to be predicted by the model.

The 3^rd^ line can be empty or can contain variable names separated by the delimiter. The variable names should be written as one word (no spaces).

The 4^th^ line indicates independent and dependent variables (given in columns) using 1 for independent/descriptor variable and 0 for dependent/target variables. The independent variables (descriptors) should be written before the dependent variables (targets).

The 5^th^ line and the lines following it contain descriptor and target values followed by ASCII code for a single character class name to which the object belongs to (e.g. 65 = A, 66 = B, etc.) and identification number of the object."

An example of input data file is given below (the delimiter used is semicolon):

Description of the file is in the first line.

4;6

x1_name;x2_name;x3_name;x4_name;x5_name;y1_name;;

1;1;1;1;1;0;;

0;0;1;0;1;5;66;1

0;1;1;1;0;14;65;2

1;1;0;0;0;24;66;3

1;1;1;0;1;29;66;4

The same example of the input data file is given below with space used as the delimiter:

Description of the file is in the first line.

4 6

x1_name x2_name x3_name x4_name x5_name y1_name

1 1 1 1 1 0

0 0 1 0 1 5 66 1

0 1 1 1 0 14 65 2

1 1 0 0 0 24 66 3

1 1 1 0 1 29 66 4

4. How to use CPANNatNIC

Data files should be prepared as written in the section above.

The main window of the program which is used to read input data is shown in Figure 1.

Short information about the program can be obtained using Help button.

Figure 1: Main window of the program.

Certain functions are unavailable until appropriate data are read by the program or generated by the program itself. Certain functions can also become unavailable during the program execution to prevent incorrect use of the program which could produce erroneous results.

The buttons used to perform modelling with counter-propagation neural networks (CPANNs) are located below the text area used to display information in the middle of the window.

First, the user should select the delimiter used from drop-down menu on the right side of the main window. Then, the data file can be read by clicking button “Select file with objects”. “Check data values” button can be used to view imported data in a table. Editing of the values in the table is also possible (the modified data can be temporarily stored in the program and can be used for modelling).

When the data have been read from data file, the user can select an appropriate model by clicking on “Select CPANN model” button or build a new model using “Train CPANN” button. The independent and dependent variables’ data should be written in the same order as in the selected model data file.

Normalization of the independent variables is possible if the model has already been made and has normalization factors included in the model file. To build a new model and save it to a model file with normalization factors, the user should first read input data file with objects and then select check-box “Use as normalization set” and press button “Normalize current descriptor data”. In this way, the independent variables will be normalized using equation (*X_i_*-*X_average_*)/*s,* where *X_i_* is value of descriptor *X* for object *i*, *X_average_* is average of all descriptor *X* values in the dataset, and *s* is their standard deviation. When a new model is made by using the button “Train CPANN”, it will be automatically saved to the file “modelweights.unw” which will contain also normalization factors (there will be no normalization factors in the model file if the user does not use the program to normalize a dataset and build a new model using it). To normalize other datasets, which are read afterwards, with the same normalization factors, uncheck the check-box “Use as normalization set” and press button “Normalize current descriptor data” after the data are read by the program.

In case the user wish to perform leave-one out cross-validation of the model, this should be done after generating a new model with the program (using button “Train CPANN”), and cannot be done after another or the same model is read from the model file. To perform leave one out cross-validation, press button “Loo-CV of CPANN with current data”.

To get the predictions from the model, the user should press button “Make predictions for the objects”.

When the predictions have been made, the button “Draw results” will become available.

“Draw results” button should be pressed to get access to graphical interface which can be used for drawing results (“top-maps”).

**Using graphical user interface for displaying results**

The graphical user interface is shown in Figure 2.

Figure 2: Graphical user interface for displaying results.

Drop-down menu indicated in Figure 2 with number 1 is used to select the content to be displayed by the interface. The drop-down menu and the button indicated by number 2 can be used to find a specific object shown on the map in the centre. Area indicated by number 3 shows the list of dataset currently read by the program. The user can select from the list which dataset should be shown on the top-map. Use “Ctrl+left mouse click” on the item in the list to select or deselect the item. “Draw” button indicated by number 4 should be pressed to draw or refresh the top-map which will appear in the centre of the window (“grey area”).

The size of the neurons shown should be manually adjusted using slider “Adjust neuron size” if the content shown on the neurons is too small or not completely visible.

Check box “Show structures” should be selected to draw 2D structures of compounds on the map. The input text file should consist of the compounds ID number followed by compound’s SMILES:

1 O=Cc1ccc(OCCCCCC)c(OC)c1

2 O=Cc1cc(c(O)c(OC)c1[N+](=O)[O-])Br

3 O=[N+]([O-])c2ccccc2(Oc1ccc(cc1)Cl)

4 O=CNc1cccc(c1C)Cl

5 O=C(OCCCC)c1cccc(c1)C(=O)OCCCC

The SMILES of the compounds from the currently selected datasets will be temporarily saved in the program until the check-box “Show structures” is unchecked.

To properly display the structures, read the SMILES after selecting the datasets in the list indicated by number 3 in Figure 2.

Figure 3: A part of top-map as seen in the graphical user interface.

Figure 3 shows a part of top-map as it can be seen in the graphical user interface. Each blue square represents one neuron. The menu shown on the map can be accessed by right mouse button click. Using the menu, some additional options are available which are not accessible from elsewhere.

Figure 4: Neuron at position x=11 and y=17 shown in a window displaying a single neuron.

Figure 4 shows a neuron in its own window. The window can be opened by “double clicking” on the neuron on the top-map or selecting “Display selected neuron” from the menu shown in Figure 3. The content in the yellow rectangle appears after clicking the structure on the neuron and slightly moving the mouse.

Figure 5: A structure shown in a new window.

Each structure on the neuron can also be viewed in a separate window as shown in Figure 5. The window appears after a right mouse button click on the structure shown in the window on Figure 4.
